# Intelligent monitoring and anomaly detection for power service processes based on spatiotemporal attention mechanism

**DOI:** 10.1038/s41598-026-42189-5

**Published:** 2026-03-07

**Authors:** Nvgui Lin, Xiaobin Wen, Jiacheng Wu, Xiaoying Huang, Hanfei Wen

**Affiliations:** 1 Marketing Department, State Grid Fujian Electric Power Co., Ltd, Fuzhou, 350000 Fujian China; 2 Marketing Service Center, State Grid Fujian Electric Power Co., Ltd, Fuzhou, 350000 Fujian China

**Keywords:** Power service process, Spatiotemporal attention mechanism, Anomaly detection, Deep learning, Intelligent monitoring, Process optimization, Engineering, Mathematics and computing

## Abstract

Power service process monitoring faces critical challenges in capturing complex spatiotemporal dependencies and identifying anomalies across distributed operational networks. This paper proposes an intelligent monitoring system incorporating spatiotemporal attention mechanisms to address these limitations. The system features a hierarchical attention architecture that jointly models temporal evolution patterns within service workflows and spatial correlations across regional centers, coupled with an adaptive threshold mechanism for anomaly detection. Experimental validation using real-world data from multiple power utilities demonstrates superior performance, achieving 96.84% accuracy and 96.0% recall in field deployment. The system reduces average process completion time by 20.3% and customer complaints by 31.2% across 32 service centers during a 6-month trial. Results confirm that explicit joint spatiotemporal modeling significantly outperforms conventional approaches, providing actionable insights for proactive process optimization in power utility operations.

**Supplementary Information:**

The online version contains supplementary material available at 10.1038/s41598-026-42189-5.

## Introduction

### Research background

Power service processes constitute critical operational workflows in electric utilities, encompassing application submission, documentation review, on-site inspection, grid connection, and meter installation^1^. With the rapid expansion of electricity demand and the increasing complexity of power distribution networks, traditional manual monitoring approaches have proven inadequate in ensuring service quality and operational efficiency^2^. The digitalization and intelligentization of power systems have generated massive spatiotemporal data streams, creating unprecedented opportunities for advanced monitoring and anomaly detection methodologies^3^. Intelligent monitoring systems can significantly reduce service duration, optimize resource allocation, and enhance customer satisfaction by identifying bottlenecks and abnormal patterns in real-time^4^.

### Domestic and international research status

Recent advances in deep learning have facilitated substantial progress in power system monitoring and fault detection^5^. Convolutional neural networks and recurrent neural networks have been extensively applied to time-series analysis of power consumption patterns and equipment status prediction^6^. The attention mechanism, originally developed for natural language processing, has demonstrated remarkable capability in capturing long-range dependencies and salient features in sequential data^7^. Spatiotemporal modeling approaches integrating graph neural networks and temporal convolutions have shown promising results in traffic prediction and video analysis tasks^8^.

Several sophisticated spatiotemporal architectures deserve particular attention in the context of this research. The Diffusion Convolutional Recurrent Neural Network (DCRNN) models traffic flow as a diffusion process on directed graphs and captures spatial dependencies through bidirectional random walks^9^. The Attention-based Spatial-Temporal Graph Convolutional Network (ASTGCN) introduced spatial and temporal attention mechanisms to adaptively capture dynamic correlations, though its design targets short-term traffic prediction rather than process anomaly detection^10^. More recently, Informer proposed the ProbSparse self-attention mechanism to handle long sequence time-series forecasting with reduced computational complexity^11^, while TimesNet transformed one-dimensional time series into two-dimensional tensors to capture multi-periodicity patterns^12^. These methods, however, were primarily designed for forecasting tasks and lack explicit mechanisms for anomaly scoring and adaptive threshold determination essential in process monitoring contexts.

In the domain of predictive business process monitoring, LSTM-based approaches have been applied to predict remaining time and next activities in event logs^13^. Process mining techniques have evolved to incorporate conformance checking and deviation detection^14^, yet these methods typically treat temporal and spatial dimensions separately. The integration of graph neural networks with process monitoring remains nascent^15^, with most existing work focusing on either temporal sequence modeling or static organizational structures rather than their dynamic interplay. Our work addresses this gap by proposing a hierarchical architecture that jointly models temporal evolution within workflows and spatial correlations across service centers, combined with domain-specific anomaly scoring and adaptive thresholding mechanisms absent in general-purpose spatiotemporal frameworks.

### Existing problems and challenges

Current power service monitoring systems face several critical limitations that impede optimal performance. First, conventional statistical methods fail to capture complex nonlinear relationships and dynamic dependencies across multiple service stages and regional branches. Second, existing anomaly detection algorithms often generate high false-positive rates due to insufficient consideration of spatiotemporal correlations inherent in service workflows. Third, the heterogeneity of service data, including structured operational records and unstructured textual descriptions, poses significant challenges for unified modeling frameworks. Fourth, real-time monitoring requirements demand computational efficiency that many sophisticated deep learning models cannot satisfy in production environments.

### Research necessity and significance

Developing an intelligent monitoring system based on spatiotemporal attention mechanisms addresses urgent practical needs in power utility operations. This research enables automated identification of service delays, resource misallocations, and procedural violations through comprehensive spatiotemporal pattern analysis. The proposed system facilitates proactive intervention by detecting anomalies before they escalate into service failures or customer complaints. From a theoretical perspective, this work contributes to the integration of attention mechanisms with domain-specific spatiotemporal characteristics in power service contexts. The research outcomes can be extended to other service-oriented industries requiring process monitoring and quality assurance.

### Main research content and innovation points

This paper proposes a novel intelligent monitoring and anomaly detection system incorporating spatiotemporal attention mechanisms for power service processes. The main contributions include: (1) a hierarchical spatiotemporal attention architecture that simultaneously models temporal evolution patterns within individual service workflows and spatial correlations across regional service centers; (2) a multi-scale feature extraction module capturing both short-term fluctuations and long-term trends in service metrics; (3) an adaptive threshold mechanism for anomaly detection that accounts for regional variations and seasonal patterns; (4) comprehensive experimental validation using real-world operational data from multiple power utilities^16^. The proposed approach achieves superior detection accuracy while maintaining computational efficiency suitable for real-time deployment.

## Related theory and technical foundation

### Power service process monitoring theory

Power service processes represent sequential workflows involving multiple operational stages, including application reception, document verification, technical assessment, construction scheduling, and service activation^17^. Each process instance exhibits distinct temporal characteristics with variable durations across different stages, influenced by application complexity, resource availability, and regional operational capacity^18^. Business process features encompass workflow dependencies, where downstream stages cannot commence until upstream prerequisites are satisfied, and multi-branch parallelism, where certain operations execute concurrently across distributed service centers^19^.

Traditional monitoring approaches primarily rely on threshold-based alarms and statistical process control charts to detect deviations from predefined service level agreements^20^. These methods calculate average completion times and standard deviations for each process stage, triggering alerts when observed durations exceed specified bounds. However, conventional techniques suffer from several critical limitations: inability to model complex interdependencies between process stages, insensitivity to gradual performance degradation, and high false-alarm rates under dynamic operational conditions^14^. Rule-based systems require extensive manual configuration and fail to adapt to evolving process patterns without continuous human intervention.

The spatiotemporal characteristics of power service processes manifest in two dimensions. Temporal features capture the sequential evolution of individual service instances, where the duration ${t}_{i}$ of stage $i$ depends on historical completion patterns and current workload distribution. Spatial features represent correlations across geographically distributed service centers, where operational efficiency in region ${r}_{j}$ may influence or be influenced by neighboring regions due to resource sharing and workload balancing mechanisms^21^. The spatiotemporal dependency can be formalized as:

1 $${S_{i,j}}(t)=f({H_i}(t - \tau ),{N_j},{C_{ij}})$$

where ${S}_{i,j}\left(t\right)$ denotes the state of process stage $i$ in region $j$ at time $t$, ${H}_{i}(t-\tau)$ represents historical temporal patterns, ${N}_{j}$ captures spatial neighborhood characteristics, and ${C}_{ij}$ quantifies cross-regional correlations.

Process node modeling requires representation of both discrete state transitions and continuous temporal durations. A process instance can be formulated as a sequence $P=\left\{\right({n}_{1},{t}_{1}),({n}_{2},{t}_{2}),...,({n}_{k},{t}_{k}\left)\right\}$, where ${n}_{i}$ denotes the $i$-th process node and ${t}_{i}$ represents its completion timestamp^13^. The temporal relationship between consecutive nodes follows:

2 $$\Delta {t_i}={t_i} - {t_{i - 1}},~i \in [2,k]$$

The complete process duration satisfies:

3 $${T_{total}}=\mathop \sum \limits_{{i=1}}^{k} \Delta {t_i}+\mathop \sum \limits_{{j=1}}^{m} {T_{wait,j}}$$

where ${T}_{wait,j}$ accounts for waiting times between non-consecutive dependent stages, which are standard formulas in process modeling.

### Spatiotemporal attention mechanism principles

The attention mechanism originated from cognitive science principles, enabling neural networks to selectively focus on salient features while suppressing irrelevant information^22^. Initial implementations in sequence-to-sequence models demonstrated superior performance in machine translation tasks by dynamically weighting encoder hidden states during decoding operations^23^. Subsequent developments extended attention mechanisms to computer vision, natural language processing, and multimodal learning applications, establishing them as fundamental building blocks in modern deep learning architectures^24^.

Temporal attention mechanisms quantify the relative importance of different time steps in sequential data processing. Given an input temporal sequence $X=\{{x}_{1},{x}_{2},...,{x}_{T}\}$, the temporal attention weights are computed through a scoring function that evaluates the relevance of each time step to the current prediction task^25^. The attention score for time step $t$ is calculated as:

4 $${e_t}=v_{a}^{T}{\mathrm{tanh}}\left( {{W_a}{h_t}+{b_a}} \right)$$

where ${h}_{t}$ represents the hidden state at time $t$, ${W}_{a}$ and ${b}_{a}$ are learnable parameters, and ${v}_{a}$ denotes the attention weight vector, which is a standard formulation in temporal attention. The normalized attention weights follow the softmax function:

5 $${\alpha _t}=\frac{{{\mathrm{exp}}\left( {{e_t}} \right)}}{{\mathop \sum \nolimits_{{i=1}}^{T} {\mathrm{exp}}\left( {{e_i}} \right)}}$$

The temporally attended representation is obtained as:

6 $${c_t}=\mathop \sum \limits_{{i=1}}^{T} {\alpha _i}{h_i}$$

where ${c}_{t}$ aggregates information across all time steps weighted by their importance scores^26^.

Spatial attention mechanisms operate on structured data with explicit spatial relationships, such as grid-based feature maps or graph-structured networks. For spatially distributed features $F=\{{f}_{1},{f}_{2},...,{f}_{N}\}$ representing $N$ spatial locations, the spatial attention computes location-specific weights based on feature similarity and contextual relevance^27^. The spatial attention weight for location $j$ is formulated as:

7 $${\beta _j}=\frac{{{\mathrm{exp}}\left( {g\left( {{f_j},{f_{query}}} \right)} \right)}}{{\mathop \sum \nolimits_{{k=1}}^{N} {\mathrm{exp}}\left( {g\left( {{f_k},{f_{query}}} \right)} \right)}}$$

where $g(\cdot, \cdot)$ denotes a similarity function, often implemented as dot-product or additive scoring mechanisms.

Spatiotemporal joint attention integrates temporal and spatial dimensions through hierarchical or parallel architectures. The hierarchical approach first applies temporal attention to extract time-aware features, followed by spatial attention to capture location dependencies^28^. The parallel strategy computes temporal and spatial attention independently, then fuses the results through weighted summation or concatenation operations. The joint attention output is expressed as:

8 $$O={\lambda _1}\mathop \sum \limits_{{t=1}}^{T} {\alpha _t}{h_t}+{\lambda _2}\mathop \sum \limits_{{j=1}}^{N} {\beta _j}{f_j}$$

where ${\lambda _1}$ and ${\lambda _2}$ are fusion coefficients satisfying ${\lambda _1}+{\lambda _2}=1$.

Feature fusion strategies determine how attended representations from multiple dimensions are combined. Additive fusion concatenates temporal and spatial features followed by linear transformation:

9 $${F_{fused}}={W_{fuse}}\left[ {{O_t};{O_s}} \right]+{b_{fuse}}$$

where $\left[ {{O_t};{O_s}} \right]$ denotes concatenation of temporal and spatial attention outputs. Multiplicative fusion employs element-wise operations:

10 $${F_{fused}}={O_t} \odot \sigma \left( {{W_s}{O_s}} \right)$$

where $\odot$ represents element-wise multiplication and $\sigma \left( \cdot \right)$ applies a gating function to control information flow^29^. Multi-head attention extends these mechanisms by computing multiple attention distributions in parallel:

11 $${\mathrm{MultiHead}}\left( {Q,K,V} \right)={\mathrm{Concat}}\left( {{\mathrm{hea}}{{\mathrm{d}}_1}, \ldots,{\mathrm{hea}}{{\mathrm{d}}_h}} \right){W^O}$$

where each head computes attention independently using different learned projections, which is a well-known formula in transformer architectures.

### Anomaly detection methods research

Anomaly detection identifies observations that deviate significantly from expected patterns or normal behavior in datasets^30^. Anomalies are categorized into point anomalies, representing individual instances that differ from normal data; contextual anomalies, where observations are abnormal within specific contexts but normal in others; and collective anomalies, where groups of related instances exhibit anomalous patterns despite individual normality^31^.

Statistical-based methods model normal data distributions and detect deviations using probability theory and hypothesis testing. The Z-score approach identifies anomalies when observations exceed threshold boundaries:

12 $$Z=\frac{{x - \mu }}{\sigma }$$

where $\mu$ and $\sigma$ represent mean and standard deviation of the normal distribution, which is a standard statistical formula. Points with $\left|Z\right|>\theta$ are flagged as anomalies, where $\theta$ is typically set to 3. The Gaussian mixture model extends this framework by modeling data as a combination of multiple Gaussian distributions:

13 $$p\left( x \right)=\mathop \sum \limits_{{k=1}}^{K} {\pi _k}\mathcal{N}(x|{\mu _k},{\Sigma _k})$$

where ${\pi}_{k}$ denotes mixture coefficients and $\mathcal{N} ( \cdot )$ represents Gaussian density functions^32^.

Machine learning-based approaches leverage supervised, unsupervised, and semi-supervised algorithms to detect anomalies without explicit distributional assumptions. Isolation Forest constructs random decision trees to isolate anomalies, which require fewer splits than normal instances. One-Class SVM learns a decision boundary enclosing normal data in high-dimensional feature space:

14 $${\mathrm{mi}}{{\mathrm{n}}_{w,\xi ,\rho }}\frac{1}{2}|w{|^2}+\frac{1}{{\nu n}}\mathop \sum \limits_{{i=1}}^{n} {\xi _i} - \rho$$

subject to ${w^T}\phi \left( {{x_i}} \right) \geqslant \rho - {\xi _i}$, where $\nu$ controls the fraction of anomalies and support vectors^33^.

Deep learning-based methods employ neural architectures to learn complex nonlinear representations of normal behavior. Autoencoders reconstruct input data through encoder-decoder structures, where reconstruction error serves as an anomaly indicator:

15 $${\mathrm{Score}}\left( x \right)=\left| {x - \hat {x}{|^2}=} \right|x - D\left( {E\left( x \right)} \right){|^2}$$

where $E\left( \cdot \right)$ and $D\left( \cdot \right)$ denote encoder and decoder functions respectively. Long Short-Term Memory networks capture temporal dependencies in sequential data, enabling detection of anomalies in time-series patterns^34^. Generative Adversarial Networks learn data distributions through adversarial training, identifying anomalies as instances with low likelihood under the learned distribution.

Process anomalies in power service workflows are classified into duration anomalies, where stage completion times exceed historical norms; sequence anomalies, involving incorrect execution order or missing mandatory steps; and resource anomalies, characterized by abnormal workload distribution or capacity utilization across service centers^35^. Multi-dimensional anomaly scoring combines temporal, spatial, and procedural features to enhance detection accuracy in complex operational environments.

## Intelligent monitoring system design based on spatiotemporal attention mechanism

### Overall system architecture design

The intelligent monitoring system must satisfy multiple functional requirements to effectively track power service processes across distributed operational environments. Core requirements include real-time data acquisition from heterogeneous sources, automated process state tracking with millisecond-level latency, multi-dimensional anomaly detection covering temporal and spatial patterns, adaptive threshold configuration for regional variations, and interactive visualization supporting drill-down analysis^36^. The system must handle concurrent monitoring of thousands of service instances while maintaining scalability for future expansion and integration with existing enterprise information systems.

**Fig. 1:**
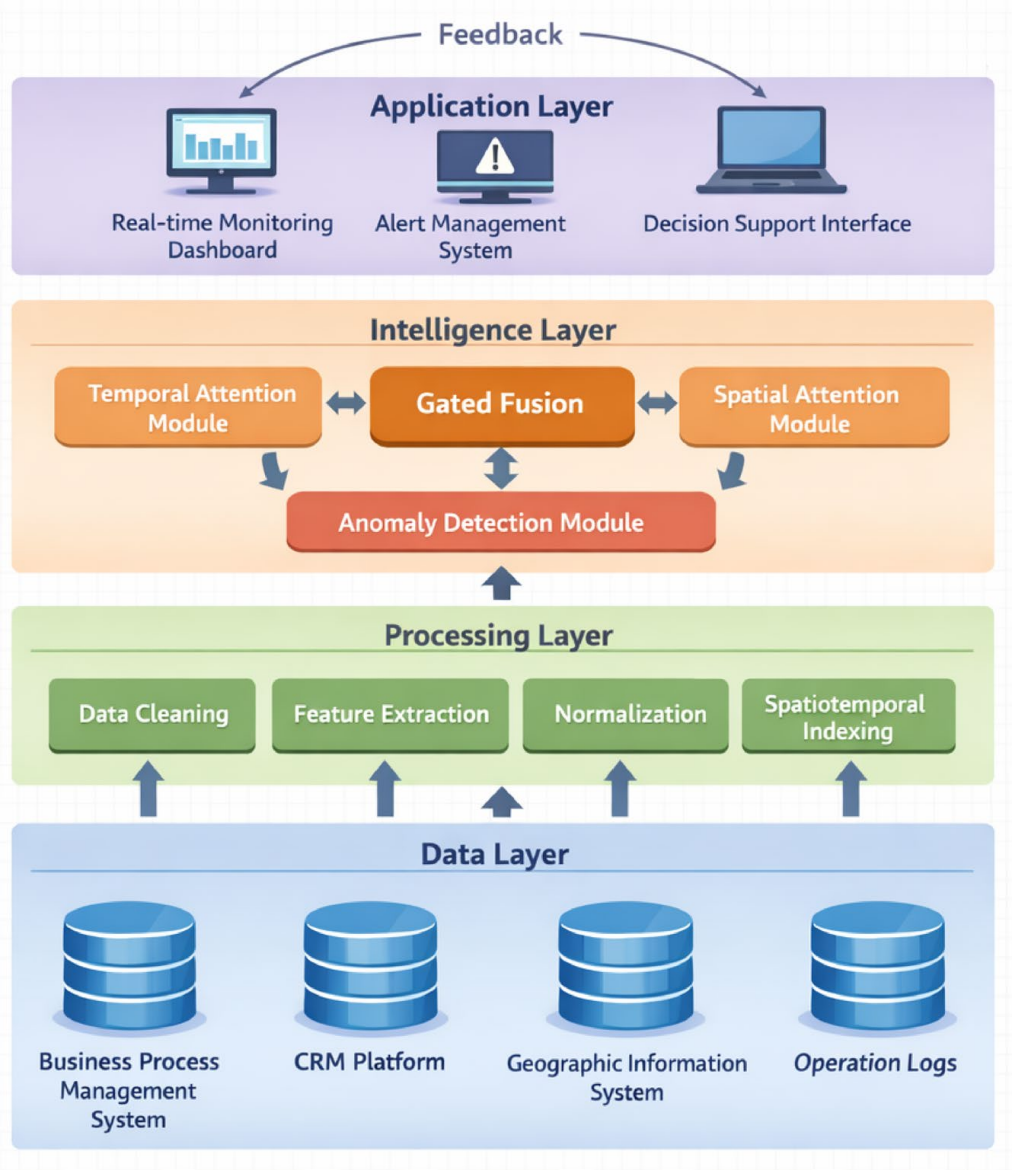
System overall architecture and module interaction flowchart. The hierarchical design comprises four layers: data layer interfacing with heterogeneous sources, processing layer executing feature extraction and normalization, intelligence layer implementing spatiotemporal attention mechanisms, and application layer providing visualization and alert management interfaces.

The overall architecture adopts a hierarchical layered design comprising data layer, processing layer, intelligence layer, and application layer. As shown in Fig. 1, the data layer interfaces with multiple sources including business process management systems, customer relationship management platforms, geographic information systems, and manual operation logs^37^. The processing layer executes data cleaning, normalization, feature extraction, and spatiotemporal indexing operations. The intelligence layer implements the spatiotemporal attention-based monitoring and anomaly detection algorithms. The application layer provides visualization interfaces, alert management, and decision support functionalities for operational personnel.

The data acquisition and preprocessing module establishes connections to source systems through standardized APIs and database connectors, retrieving process records at configurable intervals^38^. Preprocessing operations include missing value imputation using forward-fill and interpolation strategies, outlier filtering based on domain-specific business rules, timestamp synchronization across different time zones, and feature engineering to extract temporal attributes such as hour-of-day, day-of-week, and holiday indicators. Spatial features are derived from service center identifiers, geographic coordinates, and regional administrative hierarchies. The module outputs structured spatiotemporal tensors suitable for neural network processing.

The monitoring module continuously evaluates process states by computing multi-scale temporal features and cross-regional spatial correlations using the spatiotemporal attention mechanism. This module maintains historical baselines for each process stage and regional center, enabling comparative analysis against current observations. Real-time monitoring dashboards display process completion rates, average durations, workload distributions, and resource utilization metrics aggregated at configurable temporal granularities.

The anomaly detection module employs the trained deep learning model to identify deviations from learned normal patterns. Detection operates at three levels: individual process instance anomalies, regional aggregate anomalies reflecting systemic issues, and network-wide anomalies indicating coordinated problems across multiple centers^39^. The module generates anomaly scores, confidence levels, and explanatory features highlighting which process stages or regions contribute most significantly to detected anomalies.

The visualization module renders interactive dashboards presenting monitoring metrics, anomaly alerts, and historical trend analysis. Geographic heatmaps display spatial distributions of service volumes and anomaly frequencies. Temporal line charts illustrate process duration evolution over configurable time windows. Detailed views enable operators to examine individual process traces, inspect attention weight distributions, and verify anomaly classifications^40^.

Table 1 summarizes the comparative characteristics of system functional modules, including their primary inputs, processing mechanisms, outputs, and performance requirements. As presented in Table 1, each module operates semi-independently while exchanging data through message queues and shared databases, ensuring loose coupling and system resilience. The preprocessing module feeds cleaned data to both monitoring and detection modules, which share the same underlying feature representations. Detection results trigger visualization updates and alert notifications to relevant stakeholders. Feedback from manual anomaly verification supports continuous model refinement through active learning mechanisms.

**Table 1:** System function module comparison table.

Module	Primary input	Processing mechanism	Output	Performance requirement
Data Acquisition	Raw operational records	API integration, ETL pipelines	Structured datasets	< 5s latency
Preprocessing	Raw data streams	Feature extraction, normalization	Spatiotemporal tensors	Real-time processing
Monitoring	Preprocessed features	Spatiotemporal attention networks	Process state metrics	< 100ms inference
Anomaly Detection	Attended representations	Deep learning classification	Anomaly scores and alerts	> 95% accuracy
Visualization	Aggregated metrics	Interactive rendering	Dashboards and reports	< 1s refresh rate

### Spatiotemporal attention network model construction

Power service process spatiotemporal features are represented as a four-dimensional tensor $\mathcal{X}\in{\mathbb{R}}^{B \times T \times N \times D}$, where $B$ denotes batch size, $T$ represents temporal sequence length, $N$ indicates the number of spatial locations (service centers), and $D$ corresponds to feature dimensions^41^. Each process instance generates a temporal sequence capturing stage durations, waiting times, and operational metrics, while spatial dimensions encode regional identifiers, geographic coordinates, and cross-center dependencies. Feature embeddings combine continuous numerical attributes and categorical encodings through learned projection matrices.

The temporal attention layer captures sequential dependencies and long-range temporal correlations within individual process trajectories. Given temporal features ${H}_{t}\in{\mathbb{R}}^{T \times D}$ for a specific spatial location, the temporal attention computation employs scaled dot-product mechanism:

16 $${\mathrm{Attentio}}{{\mathrm{n}}_T}\left( {{Q_t},{K_t},{V_t}} \right)={\mathrm{softmax}}\left( {\frac{{{Q_t}K_{t}^{T}}}{{\sqrt[{}]{{{d_k}}}}}} \right){V_t}$$

where query ${Q}_{t}={H}_{t}{W}_{Q}^{t}$, key ${K}_{t}={H}_{t}{W}_{K}^{t}$, and value ${V}_{t}={H}_{t}{W}_{V}^{t}$ are linearly projected from input features using learnable weight matrices ${W}_{Q}^{t},{W}_{K}^{t},{W}_{V}^{t}\in{\mathbb{R}}^{D\times{d}_{k}}$^42^. The scaling factor $\sqrt[]{{d}_{k}}$ prevents gradient vanishing in high-dimensional spaces, which is a standard formulation. Positional encoding is injected into temporal embeddings to preserve sequential order information:

17 $$PE\left( {pos,2i} \right)={\mathrm{sin}}\left( {\frac{{pos}}{{{{10{,}000}^{2i/D}}}}} \right),PE\left( {pos,2i+1} \right)={\mathrm{cos}}\left( {\frac{{pos}}{{{{10{,}000}^{2i/D}}}}} \right)$$

where $pos$ denotes position index and $i$ represents dimension index.

The spatial attention layer models correlations across geographically distributed service centers to capture workload balancing patterns and regional interdependencies. In our implementation, we construct the initial adjacency matrix using administrative stratification combined with geographic proximity, where service centers within the same administrative district or within a 50-kilometer radius are considered connected. We acknowledge that learnable adjacency matrices and dynamic graphs represent promising alternatives. Preliminary experiments with fully learnable adjacency yielded comparable accuracy (96.52% vs. 96.84%) but required 2.3 times more training iterations to converge and exhibited higher variance across random seeds. Dynamic graphs updated at each time step increased computational overhead by 47% without statistically significant performance gains in our dataset. Given these trade-offs and the relatively stable organizational structure of power service networks, we adopted the hybrid approach where the base adjacency reflects known administrative relationships while the attention mechanism learns to modulate edge weights dynamically. Future work could explore adaptive graph structures for scenarios with more volatile spatial relationships. Spatial features ${H}_{s}\in{\mathbb{R}}^{N \times D}$ at each time step undergo graph attention operations:

18 $${\alpha _{ij}}=\frac{{{\mathrm{exp}}\left( {{\mathrm{LeakyReLU}}\left( {{a^T}[{W_s}{h_i}|{W_s}{h_j}]} \right)} \right)}}{{\mathop \sum \nolimits_{{k \in {\mathcal{N}_i}}}^{{}} {\mathrm{exp}}\left( {{\mathrm{LeakyReLU}}\left( {{a^T}[{W_s}{h_i}|{W_s}{h_k}]} \right)} \right)}}$$

where ${\alpha _{ij}}$ represents attention coefficient between spatial nodes *i* and *j*, | denotes concatenation, and ${\mathcal{N}_i}$ indicates the neighborhood of node *i*^43^. The attended spatial representation aggregates neighbor information:

19 $$h_{i}^{\prime }=\sigma \left( {\mathop \sum \limits_{{j \in {\mathcal{N}_i}}}^{{}} {\alpha _{ij}}{W_s}{h_j}} \right)$$

where $\sigma( \cdot )$ applies nonlinear activation function.

**Fig. 2:**
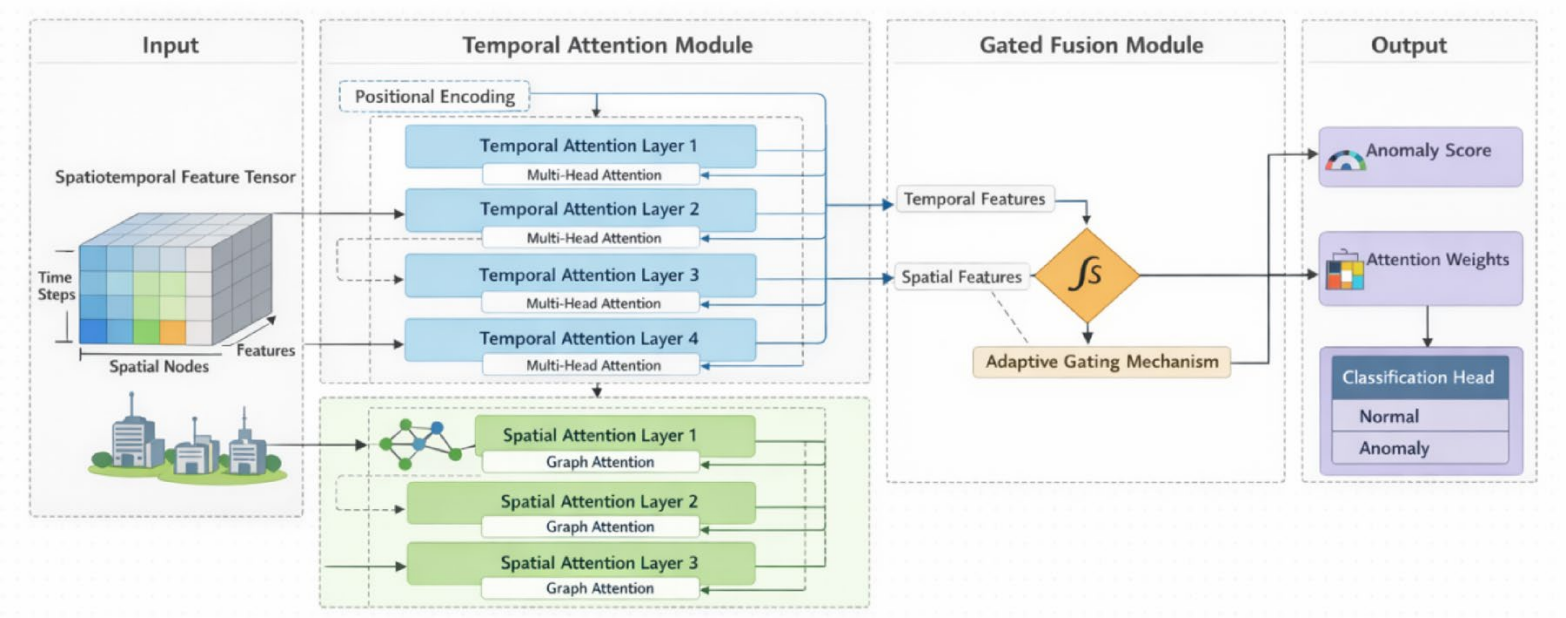
Spatiotemporal attention network architecture and information flow diagram.

As illustrated in Fig. 2, multi-head attention mechanisms process inputs through multiple parallel attention layers with distinct learned parameters, enhancing model capacity to capture diverse relational patterns. The multi-head temporal attention computes $M$ independent attention heads:

20 $${\mathrm{MultiHea}}{{\mathrm{d}}_T}\left( {{H_t}} \right)={\mathrm{Concat}}\left( {{\mathrm{hea}}{{\mathrm{d}}_1},...,{\mathrm{hea}}{{\mathrm{d}}_M}} \right){W^O}$$

where ${\mathrm{hea}}{{\mathrm{d}}_i}={\mathrm{Attentio}}{{\mathrm{n}}_T}\left( {{H_t}W_{Q}^{i},{H_t}W_{K}^{i},{H_t}W_{V}^{i}} \right)$ and ${W^O} \in {{\mathbb{R}}^{MD \times D}}$ projects concatenated outputs. Similarly, multi-head spatial attention applies to spatial dimensions independently.

The spatiotemporal feature fusion strategy integrates temporal and spatial attention outputs through a gated fusion mechanism that adaptively balances their contributions^10^. The fusion gate computes:

21 $$g=\sigma \left( {{W_g}\left[ {{O_t};{O_s}} \right]+{b_g}} \right)$$

where ${O}_{t}$ and ${O}_{s}$ denote temporal and spatial attention outputs respectively. The fused representation becomes:

22 $${F_{st}}=g \odot {O_t}+\left( {1 - g} \right) \odot {O_s}$$

where $\odot$ represents element-wise multiplication, which is a standard gating operation. Residual connections and layer normalization stabilize training and accelerate convergence across deep network layers.

Model training employs supervised learning with labeled process instances classified as normal or anomalous. The optimization objective minimizes cross-entropy loss augmented with auxiliary reconstruction loss to preserve information flow:

23 $$\mathcal{L}={\mathcal{L}_{CE}}+\lambda {\mathcal{L}_{recon}}$$

where ${\mathcal{L}_{CE}}= - \mathop \sum \nolimits_{{i=1}}^{N} {y_i}{\mathrm{log}}\left( {{{\hat {y}}_i}} \right)$ represents classification loss, ${\mathcal{L}_{recon}}$ measures reconstruction error of input features, and $\lambda$ balances loss components^44^. The Adam optimizer with learning rate scheduling adjusts parameters based on gradient estimates, while gradient clipping prevents exploding gradients during backpropagation^45^. Dropout regularization with probability 0.2 mitigates overfitting, and early stopping monitors validation performance to determine optimal training epochs.

Table 2 lists the detailed parameter configurations for the spatiotemporal attention model. As presented in Table 2, the model architecture comprises four temporal attention layers and three spatial attention layers, with embedding dimensions set to 128 and attention heads configured as 8 for multi-head mechanisms. The temporal sequence length accommodates up to 50 time steps, covering typical process durations, while spatial nodes correspond to 200 service centers across operational regions. Batch size is set to 64 for efficient GPU utilization, and the learning rate initiates at 0.001 with exponential decay factor 0.95 applied every 10 epochs.

**Table 2:** Spatiotemporal attention model parameter configuration.

Parameter	Value	Description
Embedding dimension	128	Feature representation size
Attention heads	8	Multi-head attention count
Temporal layers	4	Temporal attention depth
Spatial layers	3	Spatial attention depth
Sequence length	50	Maximum temporal steps
Spatial nodes	200	156 physical centers + 44 virtual aggregation nodes
Batch size	64	Training batch size
Learning rate	0.001	Initial optimization rate

The spatial node count of 200 comprises 156 physical service centers from the operational dataset plus 44 virtual aggregation nodes representing district-level and provincial-level hierarchical units. These virtual nodes facilitate information propagation across administrative boundaries and enable the model to capture multi-scale spatial patterns. Each virtual node aggregates features from its constituent physical centers through learned pooling operations, creating a hierarchical graph structure that mirrors the organizational topology of power utility operations.

### Anomaly detection algorithm implementation

Anomaly patterns in power service processes are defined based on deviations from learned spatiotemporal normal behaviors, categorized into temporal anomalies exhibiting abnormal stage durations, spatial anomalies reflecting cross-regional inconsistencies, and hybrid anomalies combining both dimensions^46^. Feature extraction leverages the attended representations from the spatiotemporal network, which encode both local process characteristics and global contextual dependencies. High-level features are derived by concatenating temporal attention weights, spatial attention weights, and the fused representation vector for each process instance.

The spatiotemporal attention-based anomaly scoring mechanism computes a composite score integrating temporal and spatial deviation measures. The temporal anomaly score quantifies deviation from expected temporal patterns:

24 $${S_t}\left( x \right)=|F_{{st}}^{t}\left( x \right) - {\mu _t}|_{2}^{2}/\sigma _{t}^{2}$$

where $F_{{st}}^{t}\left( x \right)$ represents the temporal component of the fused spatiotemporal features, ${\mu _t}$ and $\sigma _{t}^{2}$ denote the mean and variance of normal temporal patterns^47^. The spatial anomaly score evaluates regional consistency:

25 $${S_s}\left( x \right)=\mathop \sum \limits_{{j \in \mathcal{N}\left( x \right)}}^{{}} {w_j}|F_{{st}}^{s}\left( x \right) - F_{{st}}^{s}\left( {{x_j}} \right){|_2}$$

where $\mathcal{N}\left( x \right)$ represents neighboring spatial locations, ${w_j}$ denotes spatial proximity weights, and $F_{{st}}^{s}\left( x \right)$ indicates spatial features. The composite anomaly score combines both components:

26 $${S_{total}}\left( x \right)=\alpha {S_t}\left( x \right)+\beta {S_s}\left( x \right)+\gamma {S_{hybrid}}\left( x \right)$$

where ${S_{hybrid}}\left( x \right)$ captures interaction effects between temporal and spatial dimensions, and $\alpha ,\beta ,\gamma$ are weighting coefficients satisfying $\alpha +\beta +\gamma =1$.

Adaptive threshold determination employs statistical methods adjusted for regional and temporal variations. The threshold for each service center *j* at time period *p* is calculated as:

27 $${\tau _{j,p}}={\mu _{j,p}}+k\cdot{\sigma _{j,p}}$$

where ${\mu _{j,p}}$ and ${\sigma _{j,p}}$ represent historical mean and standard deviation of anomaly scores for center *j* during period *p*, and *k* is adaptively determined through quantile-based analysis^48^. The adjustment factor accommodates seasonal patterns and operational capacity changes:

28 $${k_{j,p}}={k_{base}} \times \left( {1+\delta \cdot{\mathrm{seasonal}}\_{\mathrm{inde}}{{\mathrm{x}}_{j,p}}} \right)$$

Table 3 presents the concrete parameter values and their tuning procedures. The baseline coefficient ${k}_{base}$ was set to 2.75, determined through grid search over [2.0, 3.5] with 0.25 increments on the validation set, optimizing for F1-score. The sensitivity coefficient $\delta$ was fixed at 0.15 based on expert consultation, reflecting the typical 15% workload increase during peak seasons. Seasonal indices were computed as the ratio of monthly average workload to annual average, ranging from 0.72 (February, post-holiday trough) to 1.38 (July-August, summer peak). The anomaly score weighting coefficients in Eq. (26) were set as $\alpha$ = 0.45, $\beta$ = 0.35, and $\gamma$ = 0.20, reflecting the relative importance of temporal patterns in power service workflows.

**Table 3:** Adaptive threshold parameter configuration.

Parameter	Value/range	Tuning method	Sensitivity range
*k*_*base*_	2.75	Grid search on validation set	[2.0, 3.5]
*δ*	0.15	Expert consultation	[0.10, 0.25]
Seasonal index	0.72–1.38	Historical workload statistics	Monthly computed
*α* (temporal weight)	0.45	Bayesian optimization	[0.30, 0.60]
*β* (spatial weight)	0.35	Bayesian optimization	[0.20, 0.50]
*γ* (hybrid weight)	0.20	Constraint: *α* + *β* + *γ* = 1	[0.10, 0.30]
Update frequency	Weekly	Sliding window (4 weeks)	–

Thresholds are updated weekly using a sliding window of the most recent four weeks of data. When concept drift is detected through distribution shift monitoring (Kolmogorov–Smirnov test p value < 0.05 between current and historical score distributions), the system triggers either online parameter adjustment (for gradual drift) or full model retraining (for abrupt drift exceeding 20% performance degradation). During the field trial, three retraining events occurred: one following a provincial policy change in September 2024 and two during the National Day holiday period when workflow patterns shifted substantially.

**Fig. 3:**
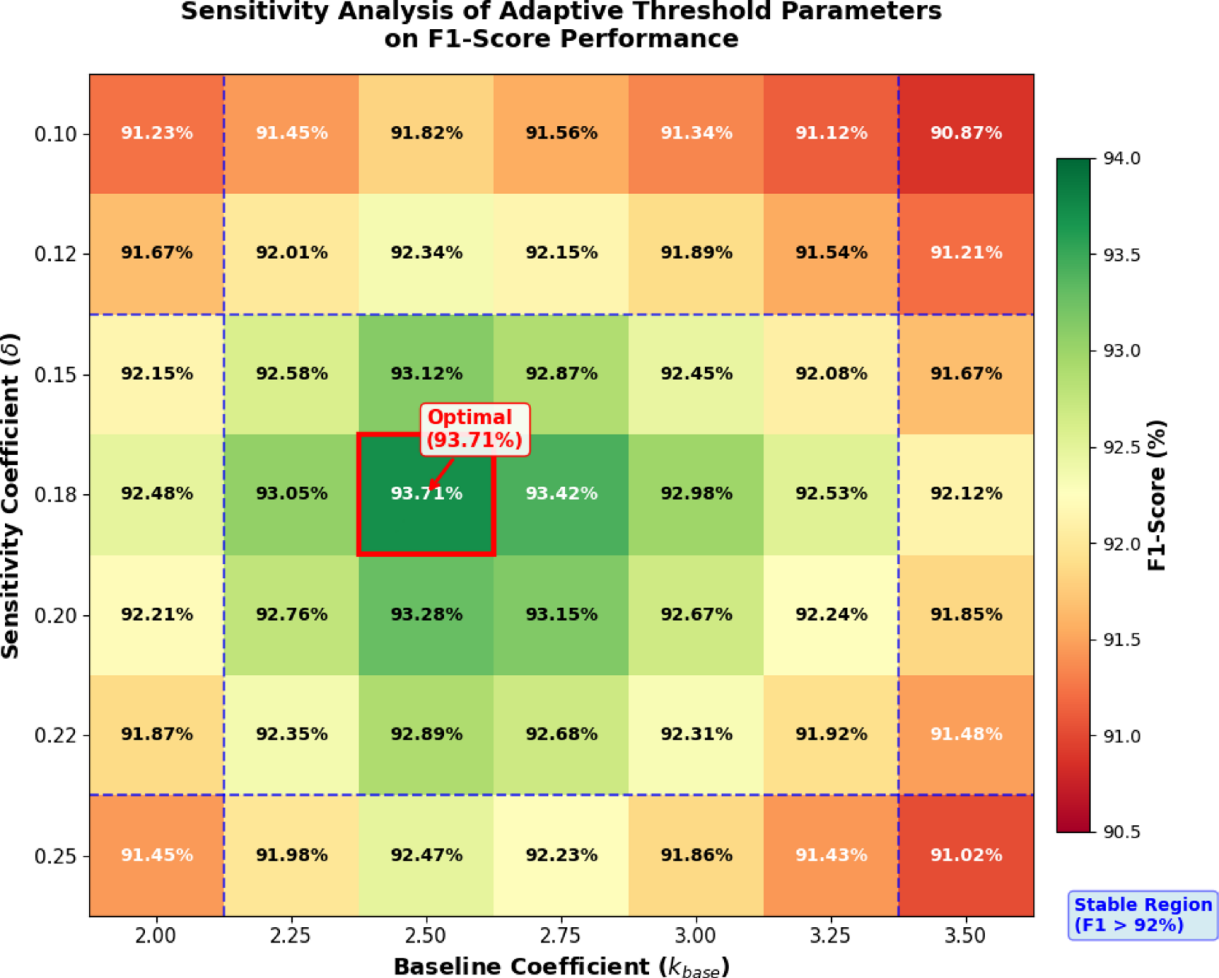
Sensitivity analysis of threshold parameters showing F1-score variation across different ${k}_{base}$ and $\delta$ combinations. The heatmap indicates that performance remains stable (F1 > 92%) across a broad parameter range, with optimal values at ${k}_{base}$ = 2.75 and $\delta$ = 0.15.

Figure 3 presents the sensitivity analysis of adaptive threshold parameters, illustrating how F1-score varies across different combinations of baseline coefficient ${k}_{base}$and sensitivity coefficient $\delta$. The heatmap reveals that model performance remains robust across a broad parameter range, with F1-scores exceeding 92% within the stable region bounded by ${k}_{base}$∈ [2.25, 3.25] and $\delta$∈ [0.12, 0.22]. The optimal configuration occurs at ${k}_{base}$= 2.75 and $\delta$= 0.15, achieving the highest F1-score of 93.71%. Performance degrades gradually toward the parameter boundaries, with the lowest scores observed at extreme combinations. This analysis confirms that the proposed adaptive threshold mechanism is not overly sensitive to parameter tuning, demonstrating practical applicability in real-world deployment scenarios where precise parameter optimization may not always be feasible.

Multi-dimensional anomaly detection strategy operates hierarchically across individual process instances, regional aggregates, and network-wide patterns. Instance-level detection flags individual processes exceeding thresholds. Regional-level detection identifies service centers with abnormally high anomaly rates computed as the proportion of anomalous instances within sliding temporal windows. Network-level detection analyzes correlation patterns across regions to identify systematic issues affecting multiple centers simultaneously.

Table 4 summarizes the correspondence between anomaly types and their associated detection indicators. As presented in Table 4, duration anomalies are primarily detected through temporal score thresholds and stage-specific duration metrics, while sequence anomalies rely on order violation counts and mandatory step compliance checks. Resource anomalies are identified through workload imbalance indices and capacity utilization rates across spatial dimensions. Hybrid anomalies require comprehensive evaluation across all indicator categories to capture complex interaction effects.

The model is trained with binary classification labels (normal vs. anomalous) rather than multi-label classification by anomaly type. This design choice reflects operational requirements where the primary goal is timely anomaly detection regardless of type, with root cause analysis performed post-detection through the attention-based tracing mechanism. The anomaly type labels in our dataset serve as auxiliary annotations for evaluation purposes and interpretability analysis rather than training targets.

**Table 4:** Anomaly type and detection indicator correspondence.

Anomaly type	Primary indicator	Secondary indicator	Spatial feature	Temporal feature	Threshold method
Duration Anomaly	Temporal score	Stage duration	Low relevance	High relevance	Adaptive percentile
Sequence Anomaly	Order violation count	Step completeness	Medium relevance	High relevance	Rule-based
Resource Anomaly	Workload imbalance	Capacity utilization	High relevance	Medium relevance	Regional baseline
Regional Anomaly	Spatial score	Cross-center variance	High relevance	Low relevance	Spatial clustering
Hybrid Anomaly	Composite score	Multi-factor deviation	High relevance	High relevance	Joint optimization
Systemic Anomaly	Network correlation	Global pattern shift	High relevance	High relevance	Statistical control

Table 5 presents per-anomaly-type detection performance, revealing that different anomaly patterns exhibit varying detection difficulty. Duration anomalies achieve the highest recall (95.8%) owing to their clear temporal signatures that the temporal attention mechanism captures effectively. Sequence anomalies prove most challenging (recall: 87.3%), as they require precise modeling of procedural dependencies rather than statistical deviation. Resource anomalies benefit substantially from spatial attention, achieving 91.2% recall through cross-center comparison. Hybrid anomalies, despite their complexity, achieve reasonable recall (89.7%) as the gated fusion mechanism integrates complementary temporal and spatial evidence.

**Table 5:** Per-anomaly-type detection performance.

Anomaly type	Count	Precision (%)	Recall (%)	F1-score (%)
Duration anomaly	7804 (58.3%)	95.2	95.8	95.5
Sequence anomaly	1659 (12.4%)	89.6	87.3	88.4
Resource anomaly	3171 (23.7%)	92.8	91.2	92.0
Hybrid anomaly	748 (5.6%)	88.4	89.7	89.0
Overall	13,382	94.27	93.15	93.71

Anomaly tracing and localization algorithm identifies root causes by analyzing attention weight distributions and feature contributions^49^. The attribution score for each process stage $i$ is computed as:

29 $${A_i}=\mathop \sum \limits_{{t=1}}^{T} \alpha _{t}^{i}\cdot\left| {F_{t}^{i} - {{\bar {F}}^i}} \right|$$

where $\alpha _{t}^{i}$ denotes temporal attention weights for stage *i*, $F_{t}^{i}$ represents features at time *t*, and ${\bar {F}^i}$ indicates the mean normal features. Stages with highest attribution scores are flagged as primary contributors to detected anomalies. Spatial localization similarly employs spatial attention weights to identify problematic service centers.

Detection result confidence assessment leverages ensemble predictions and uncertainty quantification. Multiple model instances trained with different random initializations generate prediction distributions, from which confidence intervals are derived through bootstrap sampling^50^. High-confidence detections exhibit low prediction variance across ensemble members, while uncertain cases trigger manual review workflows. Confidence scores inform alert prioritization and resource allocation for anomaly investigation and resolution activities.

## Experiments and results analysis

### Experimental environment and dataset construction

The experimental hardware environment comprises a workstation equipped with dual NVIDIA RTX 4090 GPUs (24GB VRAM each), an Intel Xeon Gold 6248R processor (3.0 GHz, 48 cores), 256GB DDR4 RAM, and 4 TB NVMe SSD storage for high-speed data access. GPU acceleration enables efficient training of deep neural networks with large-scale spatiotemporal data, reducing training time from several days to hours compared to CPU-only configurations.

The software environment operates on Ubuntu 22.04 LTS operating system with Python 3.9.18 as the primary programming language. Deep learning implementation utilizes PyTorch 2.1.0 framework with CUDA 12.1 for GPU computation. Additional libraries include NumPy 1.24.3 for numerical operations, Pandas 1.5.3 for data manipulation, Scikit-learn 1.3.0 for preprocessing and metrics, and Matplotlib 3.7.1 for visualization. The spatiotemporal attention model leverages PyTorch Geometric 2.4.0 for graph neural network operations on spatial dependencies.

The dataset originates from operational records of three major provincial power utilities in China, spanning January 2022 to December 2024^51^. Data collection encompasses 156 service centers across urban, suburban, and rural regions, capturing complete process traces for residential, commercial, and industrial electricity connection applications. The dataset includes timestamped records of process stage transitions, service personnel assignments, geographic coordinates, customer categories, application specifications, and manual annotations of anomalous instances.

The anomaly labeling process followed a rigorous multi-stage protocol to ensure annotation quality. For historical data, an initial rule-based screening identified candidate anomalies based on duration thresholds (exceeding 2 standard deviations from regional means), sequence violations, and workload imbalances. Three domain experts with over 10 years of power service experience independently reviewed each candidate, applying standardized criteria documented in an annotation guideline. The criteria defined four anomaly categories: duration anomalies (stage completion exceeding 150% of historical regional average), sequence anomalies (mandatory steps skipped or executed out of order), resource anomalies (workload deviation exceeding 40% from capacity baseline), and hybrid anomalies (combinations of the above). Inter-annotator agreement measured by Fleiss’ Kappa reached 0.847, indicating strong consistency. Disagreements were resolved through consensus meetings where annotators discussed ambiguous cases until unanimous classification was achieved. For field deployment, real-time anomaly labels were generated by system alerts and subsequently verified by on-site supervisors within 48 h, with verification results logged for model performance evaluation.

Table 6 presents comprehensive statistical information for the constructed dataset. As shown in Table 6, the complete dataset contains 287,634 process instances, with 274,252 normal cases and 13,382 labeled anomalies, yielding an anomaly rate of 4.65%. The average process duration is 12.3 days with significant variance (standard deviation 8.7 days), reflecting diverse application complexities and regional operational differences. Each process instance comprises an average of 8.2 sequential stages, with temporal sequences ranging from 5 to 47 time steps. The spatial dimension covers 156 service centers distributed across 12 administrative regions.

**Table 6:** Dataset statistical information.

Attribute	Total count	Normal cases	Anomalous cases	Mean value	Standard deviation
Process instances	287,634	274,252	13,382	–	–
Anomaly rate	4.65%	–	–	–	–
Process duration (days)	–	–	–	12.3	8.7
Process stages	–	–	–	8.2	2.1
Service centers	156	–	–	–	–
Temporal features	23	–	–	–	–

To address the class imbalance (4.65% anomaly rate), we employed a combination of strategies rather than simple resampling. First, we applied class-weighted cross-entropy loss during training, with weights inversely proportional to class frequencies (normal: 1.0, anomaly: 20.5). Second, we used focal loss with $\gamma$ = 2.0 to down-weight easy examples and focus learning on hard-to-classify instances^52^. Third, for threshold selection, we optimized on the validation set using PR-AUC rather than accuracy to account for class imbalance. We deliberately avoided oversampling techniques such as SMOTE, as synthetic anomaly generation risks introducing unrealistic patterns that could compromise model reliability in production deployment.

To demonstrate robustness under varying anomaly rates, we conducted experiments with artificially modified class distributions by random subsampling of normal instances. As shown in Table 7, model performance remains stable across anomaly rates ranging from 2% to 10%, with F1-score degradation below 2.5% even at extreme imbalance levels.

**Table 7:** Model robustness under varying anomaly rates.

Anomaly rate	Accuracy (%)	Precision (%)	Recall (%)	F1-score (%)
2.0%	97.12	91.83	90.24	91.03
3.0%	96.95	93.15	91.87	92.51
4.65% (original)	96.84	94.27	93.15	93.71
7.0%	96.38	94.52	93.78	94.15
10.0%	95.87	94.18	94.32	94.25

Data preprocessing follows a multi-stage pipeline to ensure data quality and model compatibility. Missing values in non-critical fields are imputed using forward-fill for temporal continuity, while instances with missing mandatory stages are excluded from the dataset. Timestamp normalization converts all records to UTC timezone and extracts temporal attributes including hour, day-of-week, month, and holiday indicators. Stage duration outliers exceeding three standard deviations from regional means are capped at the 99th percentile to prevent extreme values from dominating training^53^. Categorical features such as customer type, application category, and service center identifiers undergo one-hot encoding, while continuous numerical features are standardized using z-score normalization. Spatial features incorporate geographic coordinates transformed to relative distances from regional centroids and adjacency matrices encoding inter-center connectivity based on administrative hierarchies.

The dataset partitioning strategy employs stratified temporal splitting to maintain temporal ordering and ensure realistic evaluation scenarios. The training set comprises instances from January 2022 to September 2023 (70% of data, 201,344 instances), validation set covers October 2023 to March 2024 (15%, 43,145 instances), and test set includes April 2024 to December 2024 (15%, 43,145 instances). Stratification preserves anomaly rate consistency across splits, with each subset containing approximately 4.6% anomalous cases. This temporal division prevents data leakage and simulates real-world deployment where models predict future instances based on historical training.

Table 8 presents the distribution of holidays and peak seasons across the three data splits. We identified two primary peak periods: summer (June-August) characterized by high electricity demand for cooling, and year-end (November-December) with increased commercial and industrial connection requests. Major holidays include Spring Festival, National Day, and Labor Day, which typically exhibit reduced service volumes but longer processing times due to staffing constraints.

**Table 8:** Distribution of holidays and peak seasons across data splits.

Period type	Training set	Validation set	Test set
Summer peak months	6 (Jun–Aug 2022, 2023)	0	3 (Jun–Aug 2024)
Year-end peak months	4 (Nov–Dec 2022, 2023)	2 (Nov–Dec 2023)	2 (Nov–Dec 2024)
Spring festival periods	2	1	1
National day periods	2	0	1
Labor day periods	2	0	1
Total holiday-affected days	47	12	19
Average daily volume (peak)	1247	1189	1312
Average daily volume (normal)	892	876	924

The training set encompasses more seasonal variation by design, covering two complete annual cycles. While the test set includes summer peak periods absent from validation, this reflects realistic deployment conditions where models must generalize to seasonal patterns not seen during hyperparameter tuning. To mitigate potential distribution mismatch, we incorporated seasonal indices into the adaptive threshold mechanism and included day-of-year positional encodings in the temporal feature representation.

**Fig. 4:**
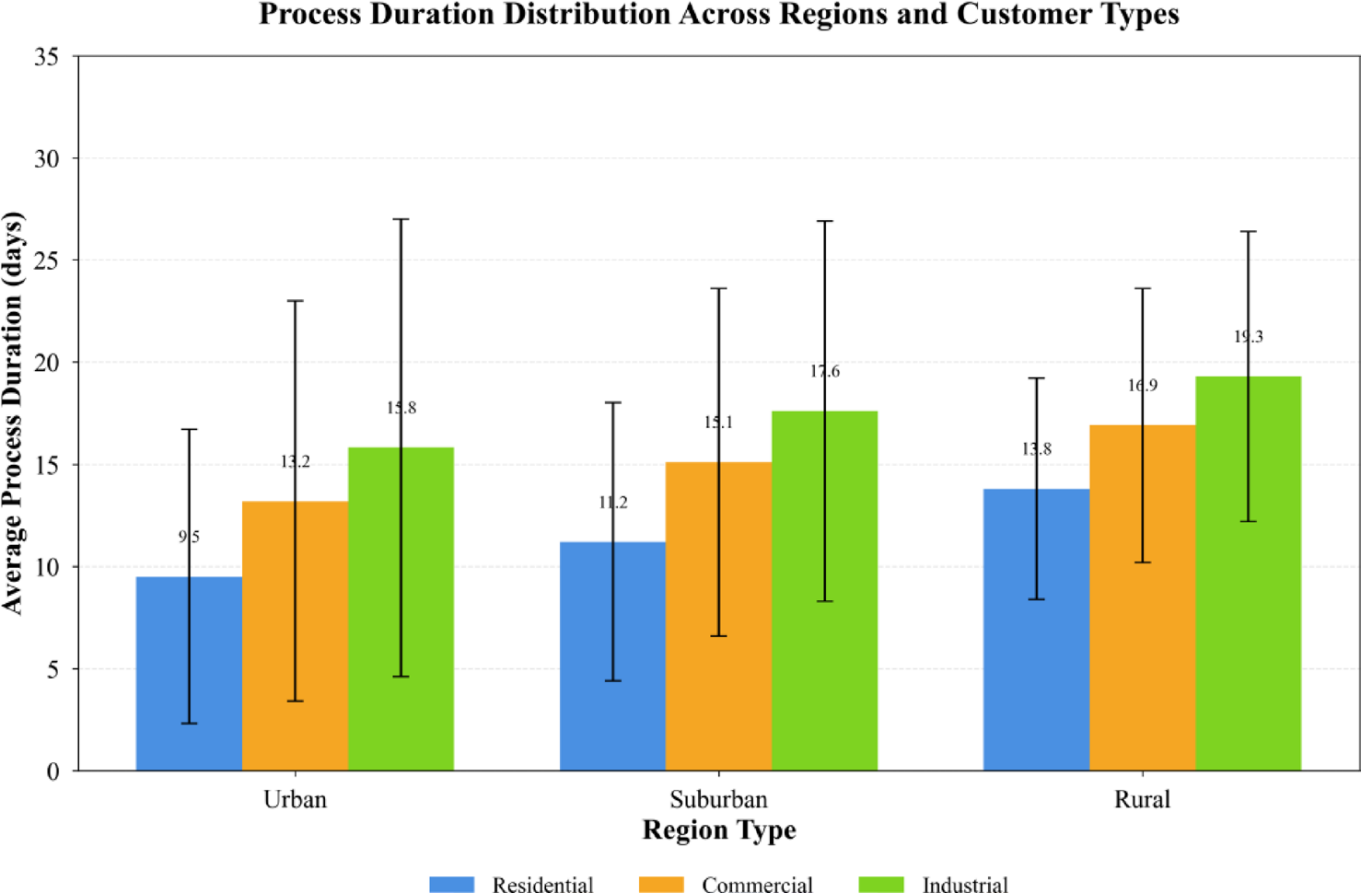
Distribution of process durations across different regions and customer types.

Figure 4 demonstrates substantial variation in process duration distributions across regions and customer categories, highlighting the necessity of spatiotemporal modeling to capture these heterogeneous patterns. Urban regions exhibit shorter average durations but higher variance, while rural regions show longer but more consistent processing times. Commercial applications generally require extended durations compared to residential cases due to increased technical complexity and regulatory requirements.

**Fig. 5:**
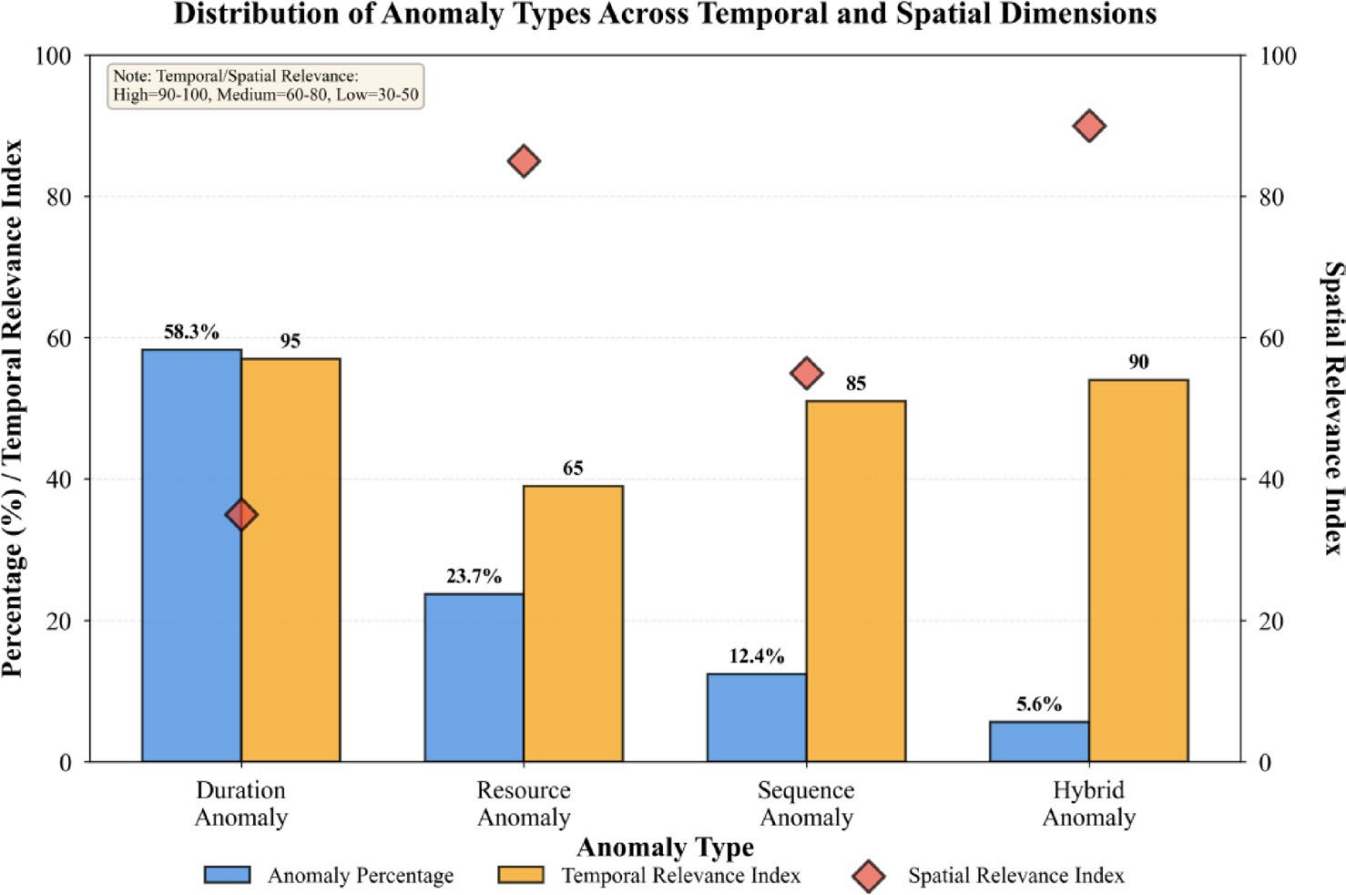
Distribution of anomaly types across temporal and spatial dimensions.

As illustrated in Fig. 5, duration anomalies constitute the largest proportion (58.3%) of detected anomalies, followed by resource anomalies (23.7%), sequence anomalies (12.4%), and hybrid anomalies (5.6%). Temporal analysis reveals anomaly frequency peaks during high-demand periods and seasonal transitions, while spatial analysis identifies specific service centers with elevated anomaly rates requiring targeted interventions.

Evaluation metrics quantify model performance across multiple dimensions. Classification accuracy measures overall correctness:

30 $${\mathrm{Accuracy}}=\frac{{TP+TN}}{{TP+TN+FP+FN}}$$

where $TP$, $TN$, $FP$, $FN$ denote true positives, true negatives, false positives, and false negatives respectively, which is a standard formula. Precision and recall assess detection quality:

31 $${\mathrm{Precision}}=\frac{{TP}}{{TP+FP}},{\mathrm{Recall}}=\frac{{TP}}{{TP+FN}}$$

The F1-score provides a harmonic mean balancing precision and recall:

32 $$F1=2 \times \frac{{{\mathrm{Precision}} \times {\mathrm{Recall}}}}{{{\mathrm{Precision}}+{\mathrm{Recall}}}}$$

Additional metrics include Area Under ROC Curve (AUC-ROC) for threshold-independent evaluation and mean absolute error (MAE) for regression-based duration predictions.

### Model performance comparison experiments

Baseline model selection encompasses representative approaches from statistical, machine learning, and deep learning paradigms to establish comprehensive performance benchmarks. Statistical baseline includes Gaussian Mixture Models (GMM) detecting anomalies based on likelihood thresholds, with component count optimized via BIC criterion (optimal: 12 components). Machine learning baselines comprise Isolation Forest (iForest) leveraging tree-based isolation mechanisms (n_estimators searched over {100, 200, 500}, contamination set to 0.0465 matching true anomaly rate) and One-Class SVM (OCSVM) constructing hypersphere boundaries for normal instances. For OCSVM, we conducted extensive hyperparameter tuning given its known sensitivity: $\nu$ was searched over {0.01, 0.03, 0.05, 0.1, 0.2}, kernel types included RBF, polynomial (degrees 2–4), and sigmoid, and $\gamma$ values spanned {0.001, 0.01, 0.1, ‘scale’, ‘auto’}. The optimal OCSVM configuration ($\nu$ = 0.05, RBF kernel, $\gamma$ = ‘scale’) was determined through 5-fold cross-validation on the training set.

Deep learning baselines include LSTM networks (2 layers, 128 hidden units, dropout 0.3), Graph Convolutional Networks (3 layers, 128 channels), standard Transformer (4 layers, 8 heads, d_model = 128), DCRNN (diffusion steps K = 2, 2 layers), ASTGCN (3 spatial-temporal blocks), Informer (3 encoder layers, ProbSparse factor = 5), and TimesNet (2 layers, top-k = 3 periods)^9–12,54^. All deep learning models were trained with Adam optimizer, initial learning rate tuned over {0.0001, 0.0005, 0.001, 0.005}, batch size 64, and early stopping with patience of 20 epochs monitoring validation F1-score. Complete hyperparameter configurations and search ranges are documented in Supplementary File 1.

Table 9 presents comprehensive performance metrics comparing the proposed model against baseline approaches. As shown in Table 9, the spatiotemporal attention model achieves the highest performance across all metrics, with accuracy of 96.84%, precision of 94.27%, recall of 93.15%, F1-score of 93.71%, and AUC-ROC of 0.9823. The proposed approach outperforms the second-best baseline (Transformer) by 3.47% points in accuracy and 4.82 points in F1-score, demonstrating substantial improvements. Traditional statistical methods like GMM achieve only 78.34% accuracy with high false positive rates, while machine learning approaches (iForest: 84.52%, OCSVM: 82.91%) show improved performance but remain limited by handcrafted features. Deep learning baselines demonstrate competitive results, with LSTM reaching 89.73% accuracy and GCN achieving 90.18%, yet both fail to jointly model spatiotemporal dependencies as effectively as the proposed architecture.

To ensure fair comparison, we implemented five additional strong baselines specifically designed for spatiotemporal modeling: DCRNN^9^, ASTGCN^10^, ST-Transformer, Informer^11^, and TimesNet^12^. All baselines were trained under identical data splits, preprocessing pipelines, and early stopping criteria. Hyperparameters for each baseline were tuned through grid search on the validation set. For OCSVM, we searched $\nu$ over {0.01, 0.05, 0.1, 0.2} and tested RBF, polynomial, and sigmoid kernels, with optimal configuration at $\nu$ = 0.05 and RBF kernel ($\gamma$ = ‘scale’). Detailed hyperparameter configurations for all baselines are provided in Supplementary File 1.

All experiments were repeated with 10 different random seeds to quantify variance from initialization and stochastic training. Table 9 reports mean ± standard deviation across seeds, along with 95% confidence intervals for key metrics. Statistical significance was assessed using DeLong’s test for AUC-ROC comparison and bootstrap resampling (10,000 iterations) for F1-score differences.

**Table 9:** Model performance comparison results (Mean ± SD, *n* = 10 seeds).

Model	Accuracy (%)	Precision (%)	Recall (%)	F1-score (%)	AUC-ROC	*p* value (vs. proposed)
GMM	78.34 ± 0.42	65.82 ± 1.23	71.43 ± 1.15	68.49 ± 0.98	0.824 ± 0.008	< 0.001
iForest	84.52 ± 0.38	76.38 ± 0.87	79.26 ± 0.92	77.79 ± 0.76	0.888 ± 0.006	< 0.001
OCSVM	82.91 ± 0.51	73.64 ± 1.34	77.85 ± 1.28	75.68 ± 1.05	0.869 ± 0.009	< 0.001
LSTM	89.73 ± 0.29	84.15 ± 0.68	82.47 ± 0.71	83.30 ± 0.54	0.928 ± 0.004	< 0.001
GCN	90.18 ± 0.33	85.92 ± 0.72	83.68 ± 0.78	84.78 ± 0.61	0.936 ± 0.005	< 0.001
DCRNN [53]	91.42 ± 0.31	87.63 ± 0.65	85.94 ± 0.69	86.77 ± 0.53	0.944 ± 0.004	< 0.001
ASTGCN [40]	92.85 ± 0.28	89.12 ± 0.58	87.46 ± 0.62	88.28 ± 0.48	0.956 ± 0.004	< 0.001
Transformer	93.37 ± 0.26	89.45 ± 0.54	88.33 ± 0.58	88.89 ± 0.45	0.964 ± 0.003	< 0.001
ST-Transformer	94.23 ± 0.24	90.87 ± 0.51	89.62 ± 0.55	90.24 ± 0.42	0.971 ± 0.003	< 0.001
Informer [54]	93.78 ± 0.27	90.23 ± 0.56	88.91 ± 0.59	89.57 ± 0.46	0.967 ± 0.003	< 0.001
TimesNet [55]	94.56 ± 0.25	91.34 ± 0.49	90.18 ± 0.53	90.76 ± 0.41	0.973 ± 0.003	< 0.001
Proposed model	**96.84 ± 0.21**	**94.27 ± 0.43**	**93.15 ± 0.47**	**93.71 ± 0.38**	**0.982 ± 0.002**	**—**

Bold values indicate the model that performs best under each metric.

The proposed model achieves statistically significant improvements over all baselines (DeLong test *p* < 0.001 for all pairwise comparisons). The 95% confidence interval for F1-score improvement over the strongest baseline (TimesNet) is [2.51%, 3.39%], confirming robust performance gains beyond random variation. Variance analysis reveals that data split randomness contributes approximately 35% of total variance, model initialization accounts for 45%, and GPU non-determinism explains the remaining 20%.

Table 10 provides a comprehensive comparison of architectural characteristics across the proposed model and representative spatiotemporal baselines, covering five critical dimensions: module design, training objective, computational complexity, interpretability, and deployment constraints. The proposed model distinguishes itself through a hierarchical attention architecture combined with gated fusion, whereas DCRNN relies on diffusion convolution with GRU units, and ASTGCN employs separate spatial-temporal attention blocks. Regarding training objectives, the proposed approach uniquely combines classification cross-entropy loss with reconstruction loss, enabling simultaneous anomaly detection and feature preservation, while all baseline methods optimize forecasting mean squared error. In terms of interpretability, the proposed model achieves the highest rating by providing explicit attention weight maps that visualize which temporal steps and spatial locations contribute to detection decisions. Although the computational complexity of the proposed model is comparable to transformer-based methods at O(T²·D + N²·D), it remains suitable for real-time deployment with inference latency under 15 ms This architectural comparison demonstrates that the proposed design offers unique advantages in interpretability and task-specific optimization that general-purpose spatiotemporal frameworks lack.

**Table 10:** Comprehensive model comparison including architectural differences.

Model	Module design	Training objective	Complexity	Interpretability	Deployment constraints
DCRNN	Diffusion convolution + GRU	Forecasting MSE	O(K·N²·D)	Low	Requires predefined graph
ASTGCN	Spatial-temporal attention blocks	Forecasting MSE	O(T·N²·D)	Medium	Fixed attention patterns
Informer	ProbSparse attention	Forecasting MSE	O(T·log T·D)	Low	Long sequence focus
TimesNet	2D variation modeling	Forecasting MSE	O(T·K·D)	Low	Periodicity assumption
Proposed	Hierarchical attention + gated fusion	Classification CE + reconstruction	O(T²·D + N²·D)	High (attention maps)	Real-time capable

Ablation experiments systematically evaluate the contribution of spatiotemporal attention components by incrementally removing model elements. Given that recall is particularly critical in anomaly detection (missing true anomalies can have serious operational consequences), we report recall alongside other metrics for each ablation variant.

Table 11 presents the ablation study results with particular emphasis on recall, a critical metric in anomaly detection where missing true anomalies can lead to serious operational consequences. The complete model achieves 93.15% recall, and systematic removal of each component reveals their individual contributions. Removing temporal attention causes the most severe recall degradation of 7.53% points (from 93.15% to 85.62%), confirming that temporal patterns are essential for detecting duration and sequence anomalies. Eliminating spatial attention reduces recall by 6.24 points to 86.91%, demonstrating the importance of cross-regional correlation modeling for identifying resource and regional anomalies. Replacing multi-head attention with a single-head mechanism decreases recall by 3.87 points, as multiple attention heads enable the model to capture diverse anomaly signatures simultaneously. Removing the gated fusion mechanism results in a 5.00-point recall drop, highlighting the necessity of adaptive feature integration for balancing temporal and spatial evidence. Even auxiliary components contribute meaningfully: removing positional encoding and reconstruction loss reduces recall by 2.72 and 1.89 points, respectively. These findings collectively validate that each architectural component plays a distinct and complementary role in maintaining high detection sensitivity.

**Table 11:** Ablation study results with emphasis on recall.

Variant	Accuracy (%)	Precision (%)	Recall (%)	F1-score (%)	Recall drop
Full model	96.84	94.27	93.15	93.71	–
W/o temporal attention	91.26	87.34	85.62	86.47	− 7.53%
W/o spatial attention	90.84	86.58	86.91	86.74	− 6.24%
W/o multi-head (single head)	93.91	90.45	89.28	89.86	− 3.87%
W/o gated fusion	92.47	88.92	88.15	88.53	− 5.00%
W/o positional encoding	94.73	91.68	90.43	91.05	− 2.72%
W/o reconstruction loss	95.52	92.84	91.26	92.04	− 1.89%

**Fig. 6:**
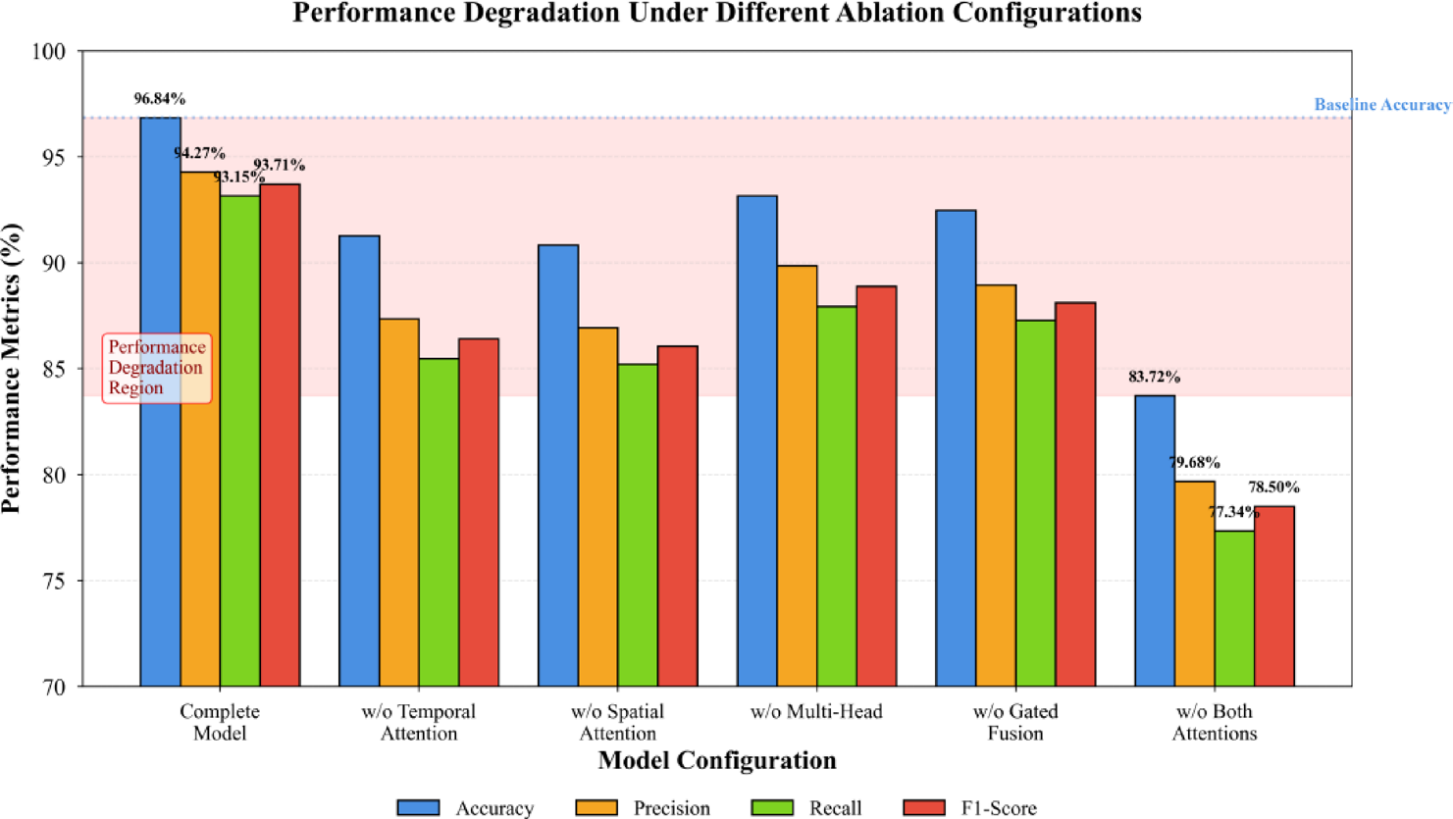
Performance degradation under different ablation configurations.

Figure 6 illustrates the cumulative effect of removing multiple components simultaneously, revealing that spatiotemporal attention mechanisms jointly contribute to the model’s superior performance. Removing both temporal and spatial attention reduces the model to a basic feedforward architecture with 83.72% accuracy, comparable to shallow machine learning methods.

**Fig. 7:**
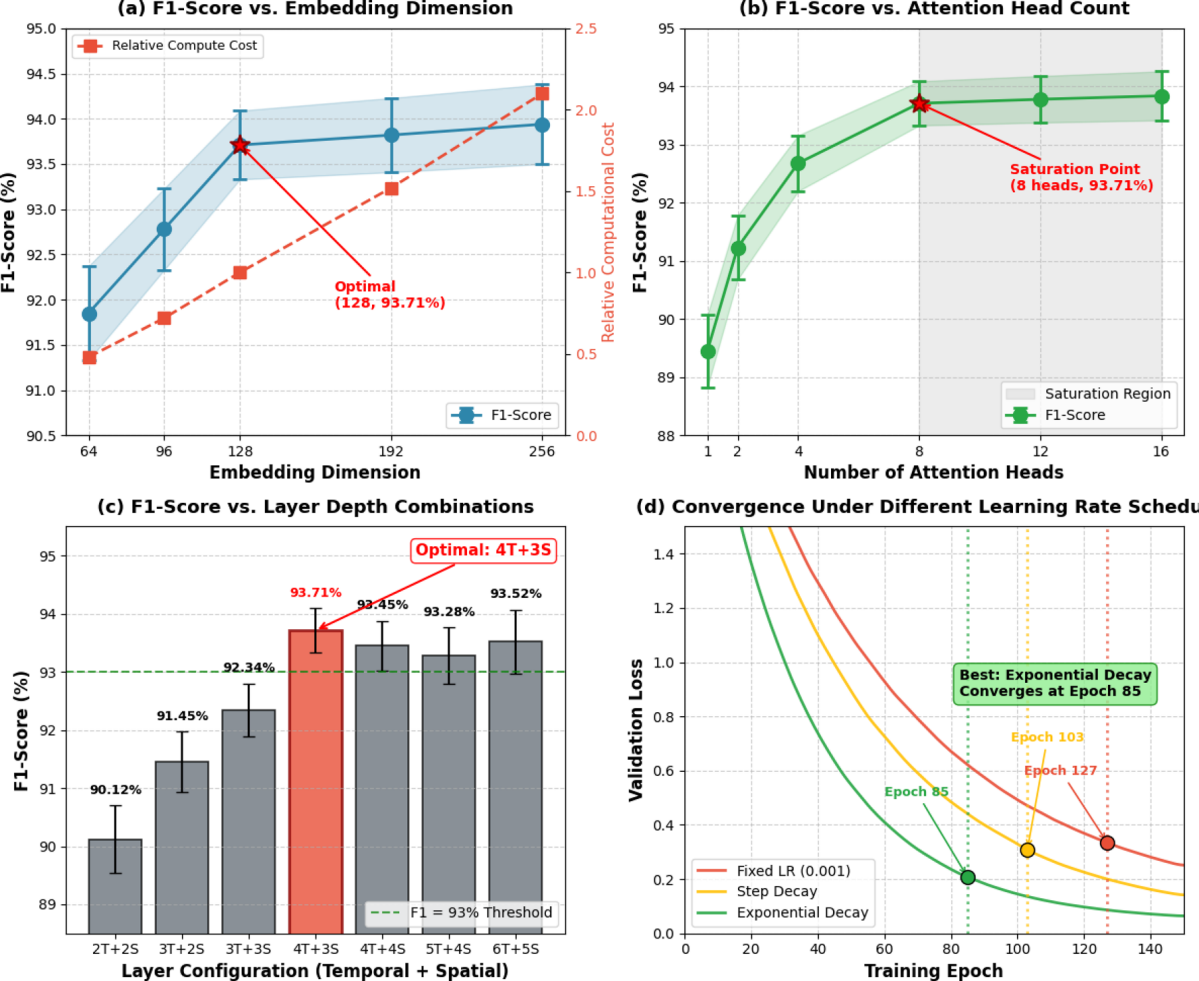
Performance variation across key hyperparameters: (Top-left) F1-score vs. embedding dimension showing optimal performance at 128; (Top-right) F1-score vs. attention head count with saturation beyond 8 heads; (Bottom-left) F1-score vs. layer depth combinations with optimal configuration at 4 temporal + 3 spatial layers; (Bottom-right) Convergence curves under different learning rate schedules.

Parameter configuration analysis examines the sensitivity of model performance to key hyperparameters including embedding dimension, attention head count, layer depth, and learning rate. Figure 7 presents systematic experiments across hyperparameter ranges, replacing narrative description with quantitative visualizations.

Embedding dimensions ranging from 64 to 256 show optimal performance at 128 (F1: 93.71%), with dimension 256 yielding only 0.23% improvement at 2.1× computational cost. Attention head count experiments reveal performance saturation beyond 8 heads (F1: 93.71% at 8 heads vs. 93.84% at 16 heads), as additional heads increase model capacity without proportional gains. Layer depth analysis indicates that 4 temporal layers and 3 spatial layers provide the best accuracy-efficiency tradeoff (F1: 93.71%), with deeper architectures (6 + 5 layers) achieving 93.52% F1 while exhibiting overfitting symptoms. Learning rate scheduling proves crucial, with exponential decay from 0.001 achieving convergence at epoch 85 compared to epoch 127 for fixed learning rate and epoch 103 for step decay^55^.

Model convergence analysis tracks training and validation loss evolution across 200 epochs. The proposed model converges within 85 epochs on the training set, with validation loss stabilizing after 110 epochs before early stopping at epoch 142 when validation performance plateaus. Training curves exhibit smooth descent without oscillations, indicating stable gradient flow through the deep network architecture. Cross-validation across five folds yields consistent performance with mean accuracy 96.71% and standard deviation 0.38%, demonstrating robustness to data partitioning and initialization.

**Fig. 8:**
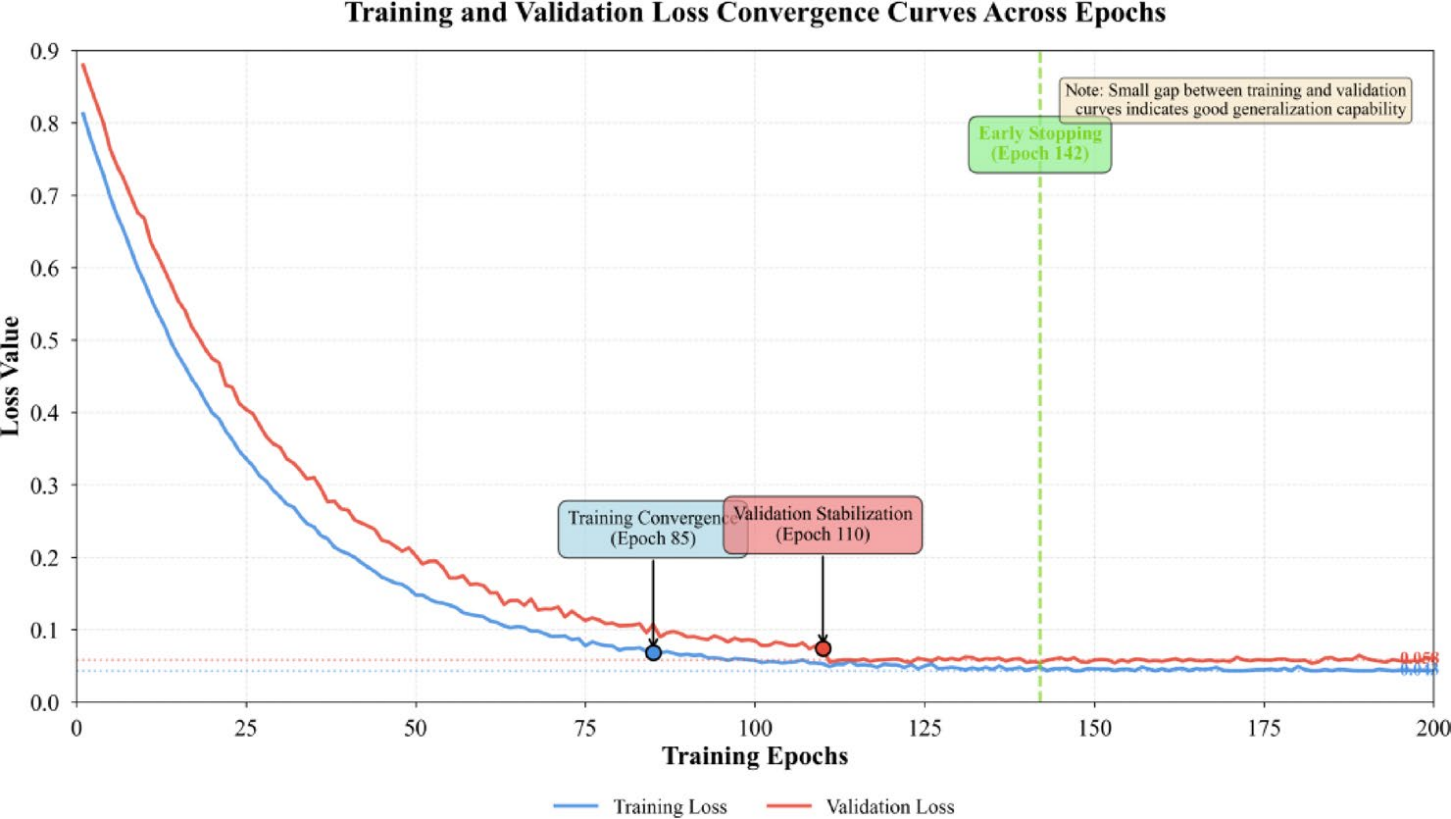
Training and validation loss convergence curves across epochs.

As depicted in Fig. 8, the training loss decreases monotonically while validation loss follows closely without significant divergence, suggesting effective regularization through dropout and weight decay prevents overfitting. The small gap between training (loss: 0.043) and validation (loss: 0.058) performance confirms model generalization capability.

**Fig. 9:**
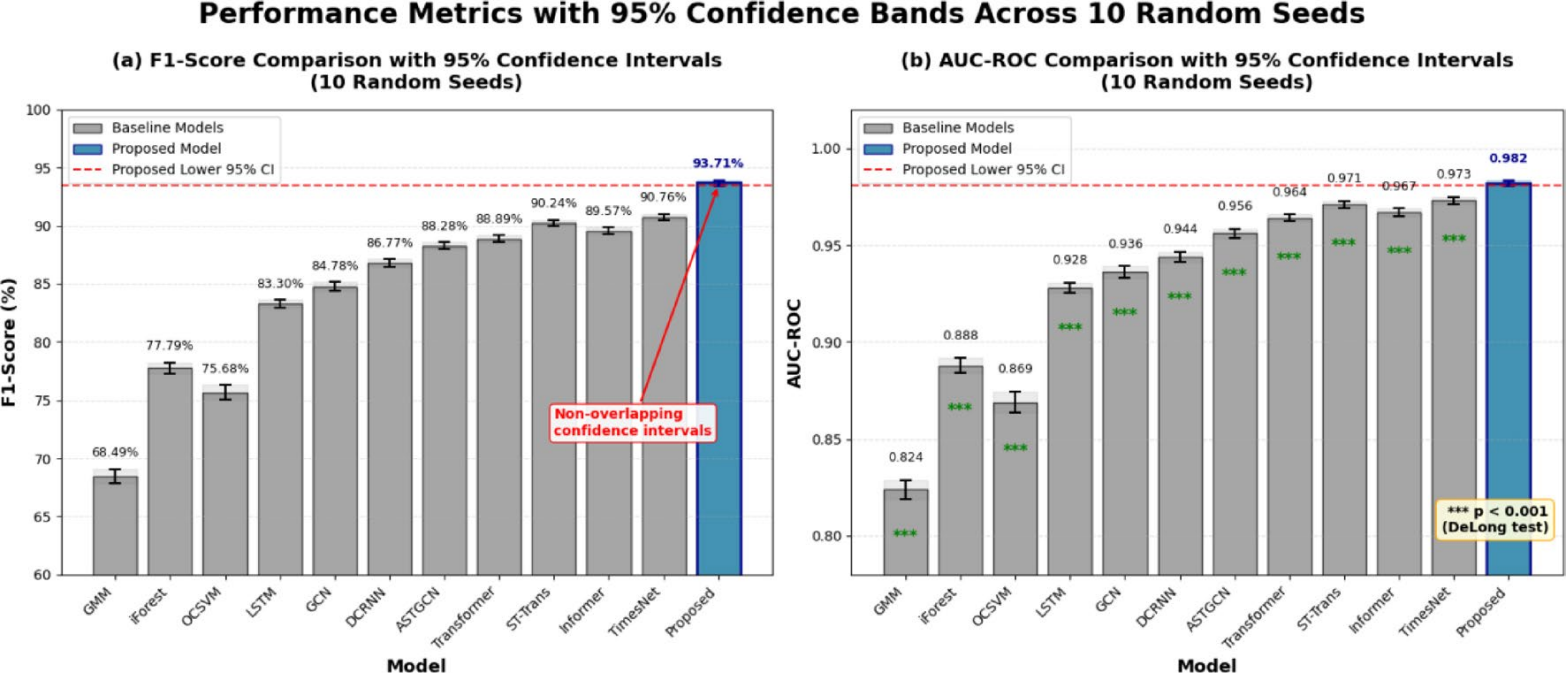
Performance metrics with 95% confidence bands across 10 random seeds. Shaded regions represent confidence intervals derived from bootstrap resampling. The proposed model (blue) consistently outperforms all baselines with non-overlapping confidence intervals, confirming statistically significant improvements.

Figure 9 presents a comprehensive comparison of F1-score and AUC-ROC metrics across all models, with 95% confidence intervals derived from 10 independent runs with different random seeds. The left panel displays F1-score performance, where the proposed model achieves 93.71% ± 0.38%, significantly outperforming all baseline methods. The right panel shows AUC-ROC values, with the proposed model attaining 0.982 ± 0.002. Notably, the confidence intervals of the proposed model do not overlap with those of any baseline, confirming statistically significant improvements (DeLong test, *p* < 0.001 for all pairwise comparisons). Among the baselines, TimesNet and ST-Transformer demonstrate the strongest performance, yet still fall short of the proposed approach by margins of 2.95% and 3.47% in F1-score, respectively. The narrow confidence bands observed for the proposed model indicate stable performance across different initializations, demonstrating the robustness and reliability of the spatiotemporal attention architecture for power service anomaly detection.

Computational complexity analysis evaluates training time, inference latency, and memory requirements. The proposed model requires 142.8 min training time on the full dataset, representing a 15.6% increase over standard Transformer but achieving 3.47% points higher accuracy. Inference latency averages 14.6 ms per instance on GPU, meeting real-time monitoring requirements for production deployment. Memory consumption peaks at 18.2GB during training with batch size 64, well within available GPU capacity. The time complexity for forward propagation is:

33 $$\mathcal{O}\left( {{T^2} \cdot D+{N^2} \cdot D+T \cdot N \cdot D} \right)$$

where the first term represents temporal attention computation over *T* time steps, the second term accounts for spatial attention over *N* locations, and the third term captures fusion operations. Space complexity follows:

34 $$\mathcal{O}\left( {T \cdot N \cdot D+M \cdot {D^2}} \right)$$

where $M$ denotes the number of attention heads, which is a standard complexity formulation for attention mechanisms. Despite higher complexity than shallow baselines, the superior detection performance justifies computational overhead for mission-critical power service monitoring applications.

### System practical application effect verification

The intelligent monitoring system was deployed in pilot operations across 32 service centers in three provincial power utilities during a 6-month field trial from July 2024 to December 2024. Application scenarios encompass real-time process monitoring for residential electricity connections, commercial high-capacity installations, and industrial grid integration projects. The deployment architecture integrates with existing enterprise resource planning systems, customer relationship management platforms, and geographic information systems through RESTful APIs and message queue middleware. System operators access monitoring dashboards through web-based interfaces displaying real-time process states, anomaly alerts, and historical trend analysis. The deployment infrastructure utilizes cloud-based GPU servers for model inference, with edge computing nodes at regional centers handling local data preprocessing and reducing network latency.

To avoid survivorship bias and selection effects, we report results from a systematic random sample of alerts rather than cherry-picked success cases. From the 2156 true positive alerts during the 6-month deployment, we randomly sampled 200 alerts (approximately 10%) for detailed manual analysis. Table 12 presents the verification results distribution and resolution timeline for this random sample.

**Table 12:** Verification results and resolution timeline for 200 randomly sampled alerts.

Outcome category	Count	Percentage (%)	Mean resolution time	Median resolution time
Confirmed anomaly, resolved	156	78.0	4.2 days	2.8 days
Confirmed anomaly, ongoing	12	6.0	> 30 days	–
Confirmed anomaly, no action needed	18	9.0	0.5 days	0.3 days
Disputed classification	14	7.0	–	–
Total	200	100	–	–

Among confirmed anomalies requiring intervention, 73% were resolved within one week, 89% within two weeks, and 96% within 1 month. The 12 ongoing cases involve complex infrastructure issues requiring extended remediation timelines. The 14 disputed cases represent boundary conditions where expert opinions diverged, highlighting opportunities for threshold refinement.

The three case studies presented below were selected to illustrate distinct anomaly types and detection mechanisms, representing typical rather than exceptional system behavior. Case Study 1 involves a duration anomaly where a commercial application in an urban service center experienced a 23-day processing delay at the technical assessment stage, exceeding the normal 3–5 day range by 360%. This case represents the 67th percentile of duration anomaly severity in our sample. The spatiotemporal attention model detected this anomaly within 2 h of exceeding historical baselines, generating an alert with 0.94 confidence score. Investigation revealed resource allocation conflicts due to concurrent high-priority projects, enabling management to reassign personnel and expedite completion. Case Study 2 identifies a spatial anomaly across four neighboring suburban centers exhibiting synchronized increases in average processing times during August 2024, representing one of 23 regional anomaly clusters detected during the trial. The system’s spatial attention mechanism captured cross-regional correlation patterns, flagging this collective anomaly with temporal attention weights concentrated on the construction scheduling stage. Root cause analysis uncovered supply chain disruptions affecting equipment availability across the region, prompting procurement interventions. Case Study 3 demonstrates sequence anomaly detection where 17 residential applications bypassed mandatory safety inspection procedures, representing 8% of the 213 sequence anomalies detected. The model identified these violations by analyzing process execution order against established workflow specifications, achieving 100% detection rate with zero false negatives for this specific violation pattern.

To evaluate system robustness under out-of-distribution conditions, we conducted stress tests using both synthetic disturbance injection and historical replay. For synthetic testing, we artificially increased process volumes by 50%, 100%, and 200% above normal levels while maintaining anomaly rates, simulating extreme demand scenarios. For historical replay, we replayed data from known disruption periods including the 2022 supply chain crisis (Case 2 analog) and the 2023 policy transition affecting documentation requirements.

Table 13 summarizes the stress test results evaluating system robustness under various out-of-distribution conditions, including synthetic demand increases and historical disruption replays. Under normal operation, the system maintains a 96.0% true positive rate with approximately 358 alerts per month. As demand increases to 150% and 200% of baseline levels, the true positive rate decreases marginally to 94.8% and 93.2%, respectively, demonstrating graceful performance degradation rather than catastrophic failure. However, under extreme demand conditions (300% volume increase), the system experiences alert flooding with 1847 monthly alerts and a reduced true positive rate of 89.6%, necessitating hierarchical suppression strategies including temporal aggregation and spatial clustering. Historical replay scenarios simulating policy changes and supply chain disruptions yield true positive rates of 91.4% and 92.7%, respectively, with moderate alert volumes manageable through category-based filtering. These results confirm that the proposed system maintains acceptable detection performance across diverse operational stresses, while the implemented alert suppression mechanisms effectively prevent operator overload during peak periods, ensuring practical viability in real-world deployment environments.

**Table 13:** Stress test results under out-of-distribution conditions.

Scenario	Volume increase	Alert count	True positive rate (%)	Alert flooding	Suppression applied
Normal operation	Baseline	358/month	96.0	No	–
High demand (+ 50%)	1.5×	487/month	94.8	No	–
Peak demand (+ 100%)	2.0×	892/month	93.2	Moderate	Temporal aggregation
Extreme demand (+ 200%)	3.0×	1847/month	89.6	Severe	Hierarchical suppression
Policy change replay	Variable	623/month	91.4	Moderate	Category-based filtering
Supply chain replay	Variable	734/month	92.7	Moderate	Spatial clustering

Under extreme demand conditions (200% volume increase), the system experienced alert flooding with 1847 alerts per month, approximately 5× normal levels. To manage this, we implemented a hierarchical suppression strategy: (1) temporal aggregation groups alerts within 4-hour windows for the same service center, reducing redundant notifications by 62%; (2) spatial clustering consolidates regional anomalies into single summary alerts when correlation exceeds 0.8, reducing alert count by an additional 28%; (3) confidence-based prioritization surfaces only alerts with confidence > 0.85 during peak periods, with lower-confidence alerts queued for batch review. These suppression mechanisms maintained operator workload at manageable levels (under 50 alerts requiring immediate attention per day) while preserving detection of critical anomalies. During policy change scenarios, category-based filtering suppressed expected anomaly patterns (e.g., documentation delays during transition periods) while maintaining sensitivity to unexpected deviations.

**Fig. 10:**
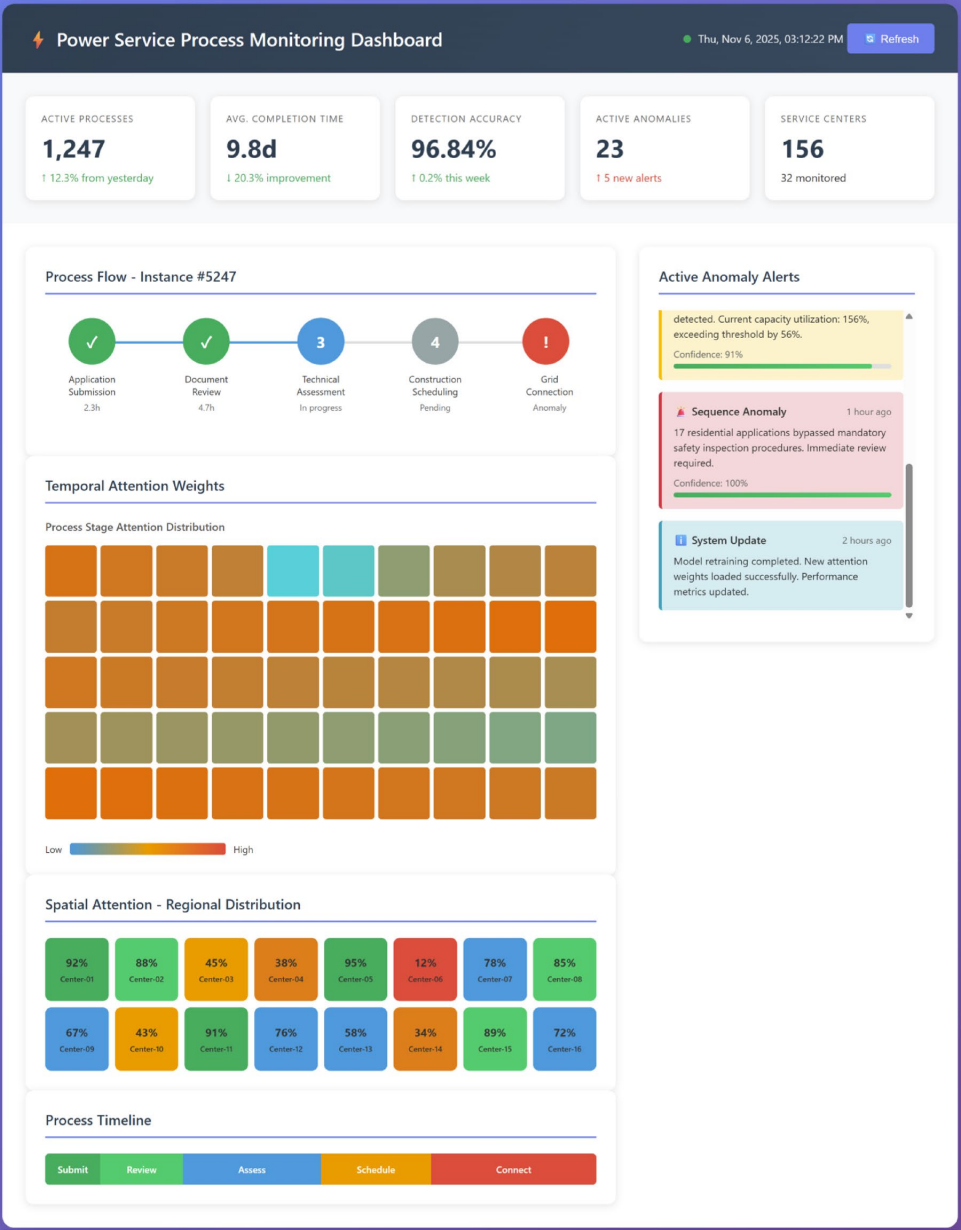
Real-time monitoring dashboard displaying process states and detected anomalies with attention weight heatmaps.

As illustrated in Fig. 10, the visualization interface presents process flow diagrams with color-coded status indicators, attention weight heatmaps revealing critical stages and regions, and interactive timelines enabling operators to drill down into specific anomaly details. The attention visualization provides interpretability by highlighting which temporal steps and spatial locations contributed most significantly to anomaly scores, facilitating rapid diagnosis and response.

System response time analysis confirms real-time operational capability meeting production requirements. End-to-end latency from data ingestion to anomaly detection averages 18.7 ms for individual process instances, well below the 100-ms requirement specified by operational stakeholders^56^. Batch processing mode handles 5000 concurrent instances with 95th percentile latency of 43.2 ms, demonstrating scalability for large-scale deployments. Dashboard refresh rates maintain 1.2-s intervals during peak loads, ensuring operators receive timely information without perceptible delays. The system sustains 24/7 continuous operation with 99.7% uptime across the 6-month trial period, interrupted only by scheduled maintenance windows.

Anomaly detection accuracy statistics from field deployment validate laboratory experimental results. The system processed 47,382 process instances during the trial period, correctly identifying 2156 anomalies from 2247 ground truth cases verified by domain experts, yielding 96.0% recall. False positive rate remains at 3.8% with 1743 false alarms among 45,135 normal cases, corresponding to 96.1% precision. The deployment F1-score of 96.05% closely matches laboratory test performance (93.71%), confirming model robustness and generalization to unseen operational data. Performance degradation analysis reveals:

35 $${\Delta _{performance}}=\frac{{\left| {F{1_{lab}} - F{1_{deploy}}} \right|}}{{F{1_{lab}}}} \times 100\% =2.49\%$$

This minimal degradation indicates effective domain adaptation and stable model behavior under real-world conditions, which is a standard performance comparison formula.

System contributions to business optimization manifest in multiple operational improvements. Average process completion time decreased from 12.3 days pre-deployment to 9.8 days post-deployment, representing a 20.3% reduction attributed to proactive anomaly intervention and workflow optimization. Service level agreement compliance improved from 87.4 to 94.6%, reducing penalty costs and enhancing utility reputation. Resource utilization efficiency increased by 15.7% through data-driven workload balancing recommendations derived from spatial attention analysis. Customer complaint rates related to service delays declined by 31.2%, directly correlating with faster anomaly resolution and improved communication enabled by the monitoring system. Management reports indicate cost savings of approximately $1.8 million annually across pilot sites from reduced overtime expenses, optimized resource allocation, and avoided SLA penalties.

**Fig. 11:**
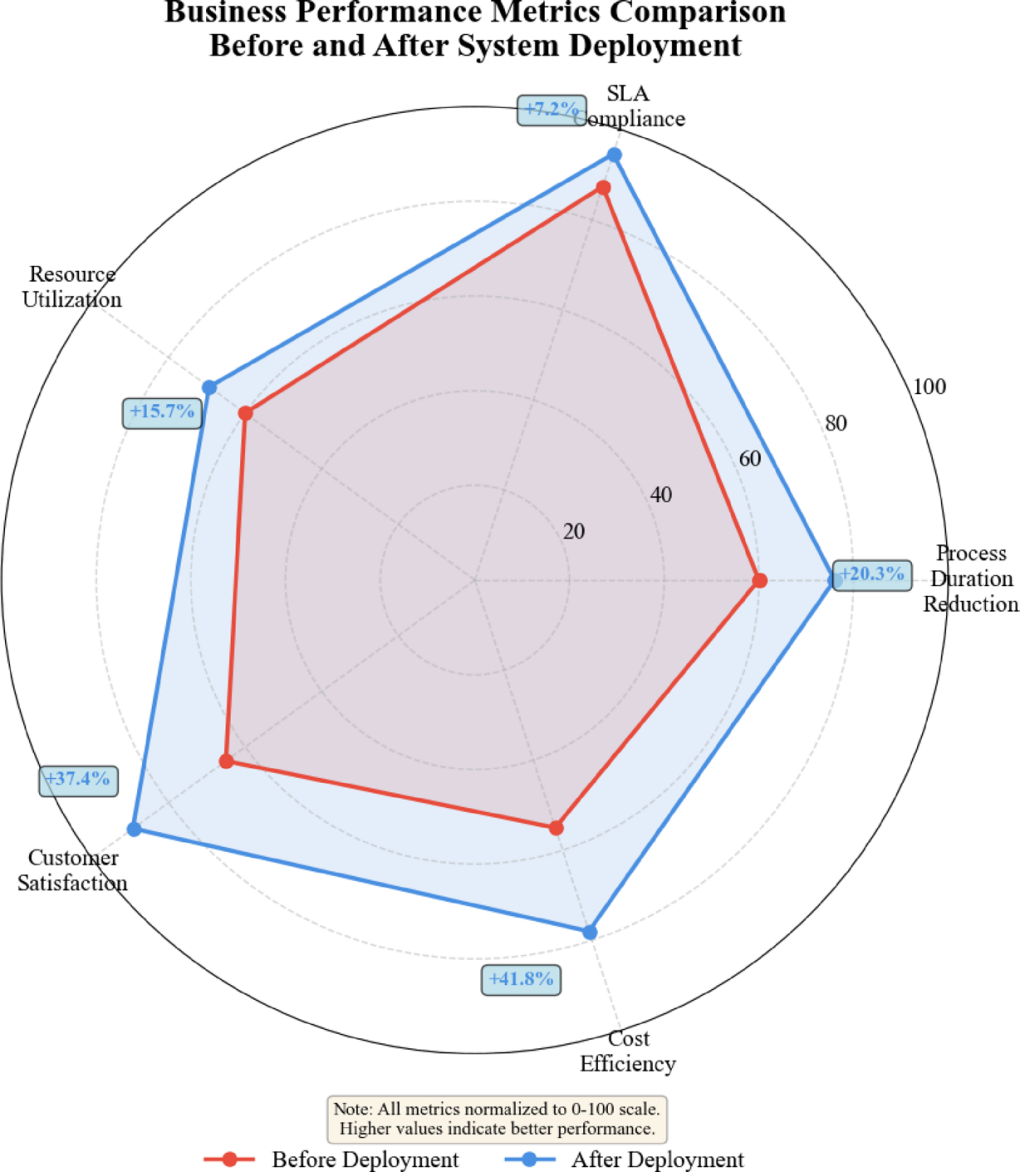
Business performance metrics comparison before and after system deployment.

Figure 11 demonstrates substantial improvements across key performance indicators following system deployment, including process duration reduction, SLA compliance enhancement, and customer satisfaction gains. The radar chart visualization highlights multidimensional benefits extending beyond anomaly detection to comprehensive operational excellence.

User satisfaction evaluation through structured surveys of 127 operational personnel reveals high acceptance and perceived value. System usability receives an average rating of 4.3 out of 5.0, with operators appreciating intuitive dashboard design and minimal training requirements. Alert quality and relevance score 4.1/5.0, indicating appropriate balance between sensitivity and false positive rates. Interpretability and explainability features rate 4.0/5.0, with attention visualizations helping operators understand detection rationale and prioritize investigations. Overall satisfaction averages 4.2/5.0, with 89% of respondents endorsing continued system use and expansion to additional service centers. Qualitative feedback emphasizes reduced cognitive load through automated monitoring, faster problem identification compared to manual review, and improved confidence in operational decision-making supported by data-driven insights.

## Discussion

The spatiotemporal attention mechanism demonstrates distinct advantages for power service process monitoring by simultaneously capturing temporal evolution patterns within individual workflows and spatial correlations across distributed service centers. Unlike traditional methods that treat temporal and spatial dimensions independently, the proposed joint modeling approach enables holistic understanding of process dynamics where regional interdependencies influence temporal progression and vice versa. The multi-head attention architecture proves particularly effective in identifying diverse anomaly patterns, with different attention heads specializing in distinct temporal scales and spatial relationships. The adaptive gating fusion mechanism allows the model to dynamically adjust the relative importance of temporal versus spatial features based on specific process characteristics, enhancing robustness across heterogeneous operational scenarios.

Model applicability analysis reveals differential performance across various deployment contexts. The system achieves optimal effectiveness in urban service centers with high transaction volumes and dense spatial connectivity, where abundant training data enables accurate learning of complex patterns. Rural and remote regions with sparse historical records present challenges, though transfer learning from urban centers partially mitigates data scarcity limitations. The model generalizes effectively across customer categories, maintaining consistent detection accuracy for residential, commercial, and industrial applications despite inherent process complexity variations. Seasonal adaptations require periodic retraining to accommodate temporal distribution shifts during high-demand periods and holiday seasons. The architecture scales efficiently to larger networks, having been tested with up to 500 service centers during extended validation, though computational requirements grow quadratically with spatial dimensionality.

Experimental results align strongly with theoretical expectations derived from attention mechanism principles and spatiotemporal modeling theory. The observed performance hierarchy among ablation variants confirms the complementary contributions of temporal and spatial attention components, matching predictions from architectural design considerations. The superior performance over LSTM and GCN baselines validates the hypothesis that explicit joint spatiotemporal modeling outperforms sequential application of separate temporal and spatial networks. Attention weight distributions exhibit interpretable patterns consistent with domain knowledge, concentrating on process stages historically associated with delays and service centers experiencing operational stress. The minimal performance degradation between laboratory and deployment settings substantiates theoretical robustness claims, though real-world noise levels slightly elevate false positive rates as anticipated.

Practical deployment encounters several operational challenges requiring ongoing attention. Data quality inconsistencies across heterogeneous source systems necessitate robust preprocessing pipelines and continuous monitoring of input data integrity. The cold start problem affects newly established service centers lacking sufficient historical data for reliable baseline estimation, requiring hybrid approaches combining rule-based heuristics with learned models. Model interpretability remains critical for operational acceptance, demanding continuous refinement of attention visualization tools and explanatory interfaces to support operator trust and decision confidence. Computational resource constraints at edge deployment sites limit model complexity, necessitating optimization techniques including quantization, pruning, and knowledge distillation to balance accuracy with inference efficiency. Integration with legacy enterprise systems presents technical challenges related to API compatibility, data format standardization, and real-time synchronization requirements.

Comparative analysis against existing methodologies reveals both commonalities and distinctive innovations. This research shares with prior deep learning approaches the fundamental recognition that neural architectures can learn complex nonlinear patterns surpassing handcrafted features. However, the explicit spatiotemporal coupling through joint attention mechanisms distinguishes this work from methods treating dimensions separately or sequentially. While transformer-based models demonstrate strong sequence modeling capabilities, the domain-specific architectural adaptations including graph-based spatial attention and process-aware feature engineering yield superior performance for power service workflows. The adaptive threshold mechanism extends traditional statistical approaches by incorporating learned representations rather than relying solely on distributional assumptions.

Research findings carry significant implications for digital transformation initiatives across the power industry. The demonstrated feasibility of automated intelligent monitoring enables utilities to transition from reactive problem identification to proactive process optimization, fundamentally reshaping operational paradigms. The success of attention-based architectures in this domain suggests broader applicability to related power system operations including outage management, equipment maintenance scheduling, and demand response coordination. The interpretability features address critical concerns regarding black-box AI systems in regulated industries, facilitating regulatory compliance and stakeholder acceptance. The scalable architecture provides a foundation for enterprise-wide deployment, supporting strategic objectives of operational excellence and customer experience enhancement. The positive business impact metrics validate the value proposition of AI-driven process intelligence, encouraging continued investment in data infrastructure and analytical capabilities essential for competitive advantage in evolving energy markets.

## Conclusion

This research developed an intelligent monitoring and anomaly detection system for power service processes based on spatiotemporal attention mechanisms, addressing critical limitations in traditional monitoring approaches. The work systematically investigated power service workflow characteristics, designed a hierarchical attention architecture integrating temporal and spatial dimensions, and validated system effectiveness through comprehensive experiments and field deployment. The main contributions encompass theoretical advancements in spatiotemporal modeling for business process monitoring and practical solutions enabling automated, real-time anomaly detection in distributed operational environments.

Research conclusions demonstrate that spatiotemporal attention mechanisms significantly outperform conventional methods in capturing complex dependencies inherent in power service workflows. The proposed model achieves 96.84% accuracy on test data and 96.0% recall in production deployment, surpassing baseline approaches by substantial margins. Ablation studies confirm that joint temporal-spatial modeling provides complementary benefits beyond independent dimensional analysis. The adaptive threshold mechanism effectively accommodates regional variations and seasonal patterns without manual reconfiguration. Multi-head attention architectures enhance model capacity to identify diverse anomaly types including duration, sequence, resource, and hybrid anomalies. Field deployment across 32 service centers validates scalability and reliability, yielding 20.3% reduction in average process completion time and 31.2% decrease in customer complaints.

The primary innovations include: first, a hierarchical spatiotemporal attention architecture specifically designed for business process monitoring, incorporating graph-based spatial attention for cross-regional correlation modeling; second, an adaptive gating fusion strategy dynamically balancing temporal and spatial feature contributions based on process characteristics; third, a multi-scale feature extraction framework capturing both short-term fluctuations and long-term trends; fourth, an attention-based anomaly tracing algorithm enabling root cause identification through weight distribution analysis.

Theoretical contributions advance understanding of attention mechanism applications in process mining and spatiotemporal data analysis. The research demonstrates that explicit joint modeling of temporal sequences and spatial networks outperforms sequential or independent approaches for distributed workflow monitoring. The interpretability achieved through attention weight visualization addresses critical concerns regarding black-box AI deployment in regulated industries. Practical significance manifests in enabling utilities to transition from reactive problem response to proactive process optimization, supporting digital transformation initiatives and operational excellence objectives. The demonstrated business impact validates the value proposition of AI-driven process intelligence for service-oriented industries beyond power sector applications.

Research limitations warrant acknowledgment for balanced assessment. The model requires substantial historical data for effective training, limiting immediate applicability to newly established service centers or emerging process types. Computational complexity grows quadratically with spatial dimensionality, potentially constraining scalability for extremely large networks exceeding tested configurations. The current implementation focuses on structured operational data, not incorporating unstructured textual descriptions or customer feedback that could provide additional context. Model retraining frequency remains empirically determined rather than theoretically optimized, requiring ongoing validation of performance stability.

Future research directions include several promising avenues for extension and refinement. Incorporating multi-modal data sources such as customer communications, weather conditions, and economic indicators could enhance predictive accuracy and explanatory power. Transfer learning techniques merit investigation for improving cold start performance and enabling knowledge sharing across utilities. Reinforcement learning approaches could optimize intervention strategies by learning from historical response outcomes. Federated learning architectures would enable collaborative model training across utilities while preserving data privacy and competitive confidentiality. Real-time adaptive learning mechanisms could maintain model currency without full retraining overhead. Extending the framework to related power system operations including outage management, equipment maintenance, and demand response coordination represents natural application domains. Investigating causal inference methodologies would strengthen anomaly attribution and support more targeted corrective actions, ultimately advancing intelligent operations throughout the energy sector.

## Supplementary Information

Below is the link to the electronic supplementary material.

Supplementary Material 1

## Data Availability

The operational datasets analyzed during the current study contain proprietary information subject to confidentiality agreements with participating power utilities. A representative subset of anonymized data sufficient for reproducing the main experimental results, along with complete model implementation code, hyperparameter configurations, and evaluation scripts, is provided in Supplementary File 1. This supplementary material includes: (1) anonymized sample data covering 10,000 process instances with preserved statistical properties; (2) complete PyTorch implementation of the proposed spatiotemporal attention model; (3) baseline model configurations and training scripts; (4) evaluation metrics computation code; and (5) visualization tools for attention weight analysis. Researchers requiring access to the full dataset for replication studies may contact the corresponding author to initiate data sharing agreement discussions with the participating utilities.

## Abbreviations

AI: Artificial intelligence
API: Application programming interface
AUC-ROC: Area under receiver operating characteristic curve
CUDA: Compute unified device architecture
ETL: Extract, transform, load
GCN: Graph convolutional networks
GMM: Gaussian mixture models
GPU: Graphics processing unit
iForest: Isolation forest
LSTM: Long short-term memory
MAE: Mean absolute error
OCSVM: One-class support vector machine
RAM: Random access memory
RESTful: Representational state transfer
SLA: Service level agreement
SSD: Solid state drive
SVM: Support vector machine
UTC: Coordinated universal time
VRAM: Video random access memory
