# Supplementary material for: Intelligent monitoring and anomaly detection for power service processes based on spatiotemporal attention mechanism
Source: Sci Rep. 2026 Mar 7;16:12479. doi: 10.1038/s41598-026-42189-5 (PMC13086843; doi:10.1038/s41598-026-42189-5)
Supplement: Supplementary file 1 — Supplementary Material 1 [file 41598_2026_42189_MOESM1_ESM.docx]

**Supplementary File 1: Intelligent Monitoring and Anomaly Detection for Power Service Processes**

**S1. Complete Hyperparameter Configurations for All Models**

**Table S1. Proposed Spatiotemporal Attention Model Hyperparameters**

| **Parameter** | **Value** | **Search Range** | **Selection Method** |
| --- | --- | --- | --- |
| Embedding dimension | 128 | {64, 128, 256, 512} | Grid search |
| Attention heads | 8 | {2, 4, 8, 16} | Grid search |
| Temporal attention layers | 4 | {2, 3, 4, 5, 6} | Grid search |
| Spatial attention layers | 3 | {2, 3, 4, 5} | Grid search |
| Dropout rate | 0.2 | {0.1, 0.2, 0.3, 0.4} | Grid search |
| Learning rate | 0.001 | {0.0001, 0.0005, 0.001, 0.005} | Grid search |
| Learning rate decay | 0.95 | {0.9, 0.95, 0.99} | Grid search |
| Decay step | 10 epochs | {5, 10, 20} | Grid search |
| Batch size | 64 | {32, 64, 128} | Memory constraint |
| Weight decay | 1e-5 | {1e-4, 1e-5, 1e-6} | Grid search |
| Gradient clip norm | 5.0 | {1.0, 5.0, 10.0} | Grid search |
| Early stopping patience | 20 | {10, 20, 30} | Validation |
| Reconstruction loss weight (λ) | 0.1 | {0.05, 0.1, 0.2} | Grid search |
| Focal loss γ | 2.0 | {1.0, 2.0, 3.0} | Grid search |
| Class weight (anomaly) | 20.5 | Inverse frequency | Computed |

**Table S2. Baseline Model Hyperparameters**

| **Model** | **Parameter** | **Value** | **Search Range** |
| --- | --- | --- | --- |
| **GMM** | n_components | 12 | {5, 8, 10, 12, 15, 20} |
|  | covariance_type | full | {full, tied, diag, spherical} |
|  | max_iter | 200 | {100, 200, 300} |
|  | reg_covar | 1e-6 | {1e-5, 1e-6, 1e-7} |
| **Isolation Forest** | n_estimators | 200 | {100, 200, 500} |
|  | max_samples | auto | {auto, 256, 512} |
|  | contamination | 0.0465 | Fixed (true rate) |
|  | max_features | 1.0 | {0.5, 0.8, 1.0} |
| **One-Class SVM** | kernel | rbf | {rbf, poly, sigmoid} |
|  | ν | 0.05 | {0.01, 0.03, 0.05, 0.1, 0.2} |
|  | γ | scale | {0.001, 0.01, 0.1, scale, auto} |
|  | degree (poly) | 3 | {2, 3, 4} |
| **LSTM** | hidden_size | 128 | {64, 128, 256} |
|  | num_layers | 2 | {1, 2, 3} |
|  | dropout | 0.3 | {0.2, 0.3, 0.4} |
|  | bidirectional | True | {True, False} |
|  | learning_rate | 0.001 | {0.0001, 0.0005, 0.001} |
| **GCN** | hidden_channels | 128 | {64, 128, 256} |
|  | num_layers | 3 | {2, 3, 4} |
|  | dropout | 0.3 | {0.2, 0.3, 0.4} |
|  | learning_rate | 0.001 | {0.0001, 0.0005, 0.001} |
| **Transformer** | d_model | 128 | {64, 128, 256} |
|  | nhead | 8 | {4, 8, 16} |
|  | num_layers | 4 | {2, 4, 6} |
|  | dim_feedforward | 512 | {256, 512, 1024} |
|  | dropout | 0.2 | {0.1, 0.2, 0.3} |
| **DCRNN** | diffusion_steps (K) | 2 | {1, 2, 3} |
|  | num_layers | 2 | {1, 2, 3} |
|  | hidden_dim | 64 | {32, 64, 128} |
|  | filter_type | dual_random_walk | {laplacian, random_walk, dual_random_walk} |
| **ASTGCN** | num_blocks | 3 | {2, 3, 4} |
|  | K (Chebyshev order) | 3 | {2, 3, 4} |
|  | nb_chev_filter | 64 | {32, 64, 128} |
|  | nb_time_filter | 64 | {32, 64, 128} |
| **ST-Transformer** | d_model | 128 | {64, 128, 256} |
|  | spatial_heads | 4 | {2, 4, 8} |
|  | temporal_heads | 4 | {2, 4, 8} |
|  | num_layers | 4 | {2, 4, 6} |
| **Informer** | d_model | 128 | {64, 128, 256} |
|  | n_heads | 8 | {4, 8} |
|  | e_layers | 3 | {2, 3, 4} |
|  | d_layers | 2 | {1, 2} |
|  | factor (ProbSparse) | 5 | {3, 5, 7} |
|  | distil | True | {True, False} |
| **TimesNet** | d_model | 128 | {64, 128, 256} |
|  | d_ff | 256 | {128, 256, 512} |
|  | num_layers | 2 | {1, 2, 3} |
|  | top_k | 3 | {2, 3, 5} |
|  | num_kernels | 6 | {4, 6, 8} |

**S2. Dataset Statistics and Sample Data Schema**

**Table S3. Detailed Feature Descriptions**

| **Feature Name** | **Type** | **Description** | **Range/Categories** |
| --- | --- | --- | --- |
| process_id | String | Unique process identifier | UUID format |
| customer_type | Categorical | Customer category | {residential, commercial, industrial} |
| application_type | Categorical | Application category | {new_connection, capacity_upgrade, relocation, temporary} |
| service_center_id | Integer | Service center identifier | 1-156 |
| region_id | Integer | Administrative region | 1-12 |
| latitude | Float | Geographic latitude | 23.5-28.5 |
| longitude | Float | Geographic longitude | 115.5-120.5 |
| stage_id | Integer | Process stage identifier | 1-12 |
| stage_name | Categorical | Process stage name | {application, document_review, technical_assessment, ...} |
| stage_start_time | Datetime | Stage start timestamp | ISO 8601 format |
| stage_end_time | Datetime | Stage end timestamp | ISO 8601 format |
| stage_duration_hours | Float | Stage duration in hours | 0.1-720.0 |
| assigned_personnel | Integer | Number of assigned staff | 1-8 |
| workload_index | Float | Current workload metric | 0.0-2.0 |
| capacity_utilization | Float | Center capacity usage | 0.0-1.0 |
| is_holiday | Binary | Holiday indicator | {0, 1} |
| day_of_week | Integer | Day of week | 0-6 |
| hour_of_day | Integer | Hour of day | 0-23 |
| month | Integer | Month | 1-12 |
| is_peak_season | Binary | Peak season indicator | {0, 1} |
| application_complexity | Float | Normalized complexity score | 0.0-1.0 |
| is_anomaly | Binary | Anomaly label | {0, 1} |
| anomaly_type | Categorical | Anomaly category | {none, duration, sequence, resource, hybrid} |

**Table S4. Process Stage Definitions**

| **Stage ID** | **Stage Name** | **Typical Duration (hours)** | **Mandatory** | **Prerequisites** |
| --- | --- | --- | --- | --- |
| 1 | Application Submission | 1-4 | Yes | None |
| 2 | Document Reception | 2-8 | Yes | Stage 1 |
| 3 | Document Verification | 4-24 | Yes | Stage 2 |
| 4 | Technical Assessment | 24-120 | Yes | Stage 3 |
| 5 | Site Survey | 8-48 | Conditional | Stage 4 |
| 6 | Safety Inspection | 4-24 | Yes | Stage 5 |
| 7 | Design Approval | 24-72 | Conditional | Stage 4 |
| 8 | Construction Scheduling | 8-48 | Yes | Stages 6, 7 |
| 9 | Installation Work | 24-168 | Yes | Stage 8 |
| 10 | Quality Verification | 4-24 | Yes | Stage 9 |
| 11 | Grid Connection | 2-8 | Yes | Stage 10 |
| 12 | Service Activation | 1-4 | Yes | Stage 11 |

**Table S5. Anonymized Sample Data Statistics (10,000 instances)**

| **Statistic** | **Value** |
| --- | --- |
| Total process instances | 10,000 |
| Normal instances | 9,535 (95.35%) |
| Anomalous instances | 465 (4.65%) |
| Duration anomalies | 271 (58.3% of anomalies) |
| Sequence anomalies | 58 (12.4% of anomalies) |
| Resource anomalies | 110 (23.7% of anomalies) |
| Hybrid anomalies | 26 (5.6% of anomalies) |
| Mean process duration (days) | 12.28 |
| Std process duration (days) | 8.64 |
| Min process duration (days) | 1.2 |
| Max process duration (days) | 67.4 |
| Service centers represented | 156 |
| Regions represented | 12 |
| Date range | 2022-01-01 to 2024-12-31 |

**S3. Model Implementation Code**

**S3.1 Spatiotemporal Attention Model (PyTorch)**

import torch

import torch.nn as nn

import torch.nn.functional as F

from torch_geometric.nn import GATConv

import math

class PositionalEncoding(nn.Module):

"""Positional encoding for temporal sequences."""

def __init__(self, d_model, max_len=100):

super(PositionalEncoding, self).__init__()

pe = torch.zeros(max_len, d_model)

position = torch.arange(0, max_len, dtype=torch.float).unsqueeze(1)

div_term = torch.exp(torch.arange(0, d_model, 2).float() * (-math.log(10000.0) / d_model))

pe[:, 0::2] = torch.sin(position * div_term)

pe[:, 1::2] = torch.cos(position * div_term)

pe = pe.unsqueeze(0)

self.register_buffer('pe', pe)

def forward(self, x):

return x + self.pe[:, :x.size(1), :]

class TemporalAttentionLayer(nn.Module):

"""Multi-head temporal attention layer."""

def __init__(self, d_model, n_heads, dropout=0.1):

super(TemporalAttentionLayer, self).__init__()

self.d_model = d_model

self.n_heads = n_heads

self.d_k = d_model // n_heads

self.W_Q = nn.Linear(d_model, d_model)

self.W_K = nn.Linear(d_model, d_model)

self.W_V = nn.Linear(d_model, d_model)

self.W_O = nn.Linear(d_model, d_model)

self.dropout = nn.Dropout(dropout)

self.layer_norm = nn.LayerNorm(d_model)

def forward(self, x, mask=None):

batch_size, seq_len, _ = x.size()

residual = x

Q = self.W_Q(x).view(batch_size, seq_len, self.n_heads, self.d_k).transpose(1, 2)

K = self.W_K(x).view(batch_size, seq_len, self.n_heads, self.d_k).transpose(1, 2)

V = self.W_V(x).view(batch_size, seq_len, self.n_heads, self.d_k).transpose(1, 2)

scores = torch.matmul(Q, K.transpose(-2, -1)) / math.sqrt(self.d_k)

if mask is not None:

scores = scores.masked_fill(mask == 0, -1e9)

attn_weights = F.softmax(scores, dim=-1)

attn_weights = self.dropout(attn_weights)

context = torch.matmul(attn_weights, V)

context = context.transpose(1, 2).contiguous().view(batch_size, seq_len, self.d_model)

output = self.W_O(context)

output = self.dropout(output)

output = self.layer_norm(output + residual)

return output, attn_weights

class SpatialAttentionLayer(nn.Module):

"""Graph attention layer for spatial dependencies."""

def __init__(self, in_channels, out_channels, heads=8, dropout=0.1):

super(SpatialAttentionLayer, self).__init__()

self.gat = GATConv(in_channels, out_channels // heads, heads=heads, dropout=dropout, concat=True)

self.layer_norm = nn.LayerNorm(out_channels)

self.dropout = nn.Dropout(dropout)

def forward(self, x, edge_index):

residual = x

out = self.gat(x, edge_index)

out = self.dropout(out)

out = self.layer_norm(out + residual)

return out

class GatedFusion(nn.Module):

"""Gated fusion mechanism for combining temporal and spatial features."""

def __init__(self, d_model):

super(GatedFusion, self).__init__()

self.W_g = nn.Linear(d_model * 2, d_model)

self.sigmoid = nn.Sigmoid()

def forward(self, temporal_feat, spatial_feat):

combined = torch.cat([temporal_feat, spatial_feat], dim=-1)

gate = self.sigmoid(self.W_g(combined))

fused = gate * temporal_feat + (1 - gate) * spatial_feat

return fused, gate

class FeedForward(nn.Module):

"""Position-wise feed-forward network."""

def __init__(self, d_model, d_ff, dropout=0.1):

super(FeedForward, self).__init__()

self.linear1 = nn.Linear(d_model, d_ff)

self.linear2 = nn.Linear(d_ff, d_model)

self.dropout = nn.Dropout(dropout)

self.layer_norm = nn.LayerNorm(d_model)

def forward(self, x):

residual = x

x = F.gelu(self.linear1(x))

x = self.dropout(x)

x = self.linear2(x)

x = self.dropout(x)

return self.layer_norm(x + residual)

class SpatiotemporalAttentionBlock(nn.Module):

"""Combined spatiotemporal attention block."""

def __init__(self, d_model, n_heads, d_ff, dropout=0.1):

super(SpatiotemporalAttentionBlock, self).__init__()

self.temporal_attn = TemporalAttentionLayer(d_model, n_heads, dropout)

self.spatial_attn = SpatialAttentionLayer(d_model, d_model, n_heads, dropout)

self.gated_fusion = GatedFusion(d_model)

self.feed_forward = FeedForward(d_model, d_ff, dropout)

def forward(self, x, edge_index, batch_size, n_nodes, seq_len):

# x shape: (batch_size, seq_len, n_nodes, d_model)

d_model = x.size(-1)

# Temporal attention: process each node's temporal sequence

x_temporal = x.permute(0, 2, 1, 3).contiguous() # (B, N, T, D)

x_temporal = x_temporal.view(batch_size * n_nodes, seq_len, d_model)

x_temporal, temporal_weights = self.temporal_attn(x_temporal)

x_temporal = x_temporal.view(batch_size, n_nodes, seq_len, d_model)

x_temporal = x_temporal.permute(0, 2, 1, 3) # (B, T, N, D)

# Spatial attention: process each time step's spatial graph

x_spatial_list = []

for t in range(seq_len):

x_t = x[:, t, :, :].contiguous().view(batch_size * n_nodes, d_model)

# Expand edge_index for batch

batch_edge_index = self._expand_edge_index(edge_index, batch_size, n_nodes)

x_t = self.spatial_attn(x_t, batch_edge_index)

x_t = x_t.view(batch_size, n_nodes, d_model)

x_spatial_list.append(x_t)

x_spatial = torch.stack(x_spatial_list, dim=1) # (B, T, N, D)

# Gated fusion

x_fused, gate = self.gated_fusion(x_temporal, x_spatial)

# Feed-forward

x_fused = x_fused.view(batch_size * seq_len * n_nodes, d_model)

x_out = self.feed_forward(x_fused)

x_out = x_out.view(batch_size, seq_len, n_nodes, d_model)

return x_out, temporal_weights, gate

def _expand_edge_index(self, edge_index, batch_size, n_nodes):

edge_indices = []

for b in range(batch_size):

edge_indices.append(edge_index + b * n_nodes)

return torch.cat(edge_indices, dim=1)

class SpatiotemporalAttentionModel(nn.Module):

"""Main spatiotemporal attention model for anomaly detection."""

def __init__(self,

input_dim,

d_model=128,

n_heads=8,

n_temporal_layers=4,

n_spatial_layers=3,

d_ff=512,

max_seq_len=50,

n_nodes=200,

n_classes=2,

dropout=0.2):

super(SpatiotemporalAttentionModel, self).__init__()

self.d_model = d_model

self.n_nodes = n_nodes

# Input embedding

self.input_embedding = nn.Linear(input_dim, d_model)

self.positional_encoding = PositionalEncoding(d_model, max_seq_len)

# Spatiotemporal attention blocks

self.st_blocks = nn.ModuleList([

SpatiotemporalAttentionBlock(d_model, n_heads, d_ff, dropout)

for _ in range(max(n_temporal_layers, n_spatial_layers))

])

# Reconstruction decoder (auxiliary task)

self.decoder = nn.Sequential(

nn.Linear(d_model, d_ff),

nn.GELU(),

nn.Dropout(dropout),

nn.Linear(d_ff, input_dim)

)

# Classification head

self.classifier = nn.Sequential(

nn.Linear(d_model, d_ff),

nn.GELU(),

nn.Dropout(dropout),

nn.Linear(d_ff, d_model),

nn.GELU(),

nn.Dropout(dropout),

nn.Linear(d_model, n_classes)

)

# Anomaly scoring heads

self.temporal_scorer = nn.Linear(d_model, 1)

self.spatial_scorer = nn.Linear(d_model, 1)

self._init_weights()

def _init_weights(self):

for module in self.modules():

if isinstance(module, nn.Linear):

nn.init.xavier_uniform_(module.weight)

if module.bias is not None:

nn.init.zeros_(module.bias)

def forward(self, x, edge_index, return_attention=False):

"""

Args:

x: Input tensor of shape (batch_size, seq_len, n_nodes, input_dim)

edge_index: Graph edge index of shape (2, num_edges)

return_attention: Whether to return attention weights

Returns:

logits: Classification logits

reconstruction: Reconstructed input (for auxiliary loss)

anomaly_scores: Dict of anomaly scores

attention_weights: (optional) Attention weight tensors

"""

batch_size, seq_len, n_nodes, input_dim = x.size()

# Input embedding

x = self.input_embedding(x)

# Add positional encoding (temporal dimension)

x_reshaped = x.permute(0, 2, 1, 3).contiguous().view(batch_size * n_nodes, seq_len, self.d_model)

x_reshaped = self.positional_encoding(x_reshaped)

x = x_reshaped.view(batch_size, n_nodes, seq_len, self.d_model).permute(0, 2, 1, 3)

# Store attention weights

all_temporal_weights = []

all_gates = []

# Process through spatiotemporal blocks

for block in self.st_blocks:

x, temporal_weights, gate = block(x, edge_index, batch_size, n_nodes, seq_len)

all_temporal_weights.append(temporal_weights)

all_gates.append(gate)

# Global pooling

x_pooled = x.mean(dim=[1, 2]) # (batch_size, d_model)

# Classification

logits = self.classifier(x_pooled)

# Reconstruction

reconstruction = self.decoder(x)

# Anomaly scores

temporal_score = self.temporal_scorer(x.mean(dim=2)).squeeze(-1) # (B, T)

spatial_score = self.spatial_scorer(x.mean(dim=1)).squeeze(-1) # (B, N)

anomaly_scores = {

'temporal': temporal_score.mean(dim=1),

'spatial': spatial_score.mean(dim=1),

'composite': 0.45 * temporal_score.mean(dim=1) + 0.35 * spatial_score.mean(dim=1) + 0.20 * (temporal_score.mean(dim=1) * spatial_score.mean(dim=1))

}

if return_attention:

return logits, reconstruction, anomaly_scores, {

'temporal_weights': all_temporal_weights,

'gates': all_gates

}

return logits, reconstruction, anomaly_scores

class FocalLoss(nn.Module):

"""Focal Loss for handling class imbalance."""

def __init__(self, alpha=None, gamma=2.0, reduction='mean'):

super(FocalLoss, self).__init__()

self.alpha = alpha

self.gamma = gamma

self.reduction = reduction

def forward(self, inputs, targets):

ce_loss = F.cross_entropy(inputs, targets, reduction='none', weight=self.alpha)

pt = torch.exp(-ce_loss)

focal_loss = ((1 - pt) ** self.gamma) * ce_loss

if self.reduction == 'mean':

return focal_loss.mean()

elif self.reduction == 'sum':

return focal_loss.sum()

return focal_loss

class CombinedLoss(nn.Module):

"""Combined loss function with classification and reconstruction components."""

def __init__(self, class_weights, gamma=2.0, recon_weight=0.1):

super(CombinedLoss, self).__init__()

self.focal_loss = FocalLoss(alpha=class_weights, gamma=gamma)

self.recon_weight = recon_weight

def forward(self, logits, targets, reconstruction, original_input):

cls_loss = self.focal_loss(logits, targets)

recon_loss = F.mse_loss(reconstruction, original_input)

total_loss = cls_loss + self.recon_weight * recon_loss

return total_loss, cls_loss, recon_loss

**S3.2 Training Script**

import torch

import torch.optim as optim

from torch.utils.data import DataLoader, Dataset

import numpy as np

from sklearn.metrics import accuracy_score, precision_score, recall_score, f1_score, roc_auc_score

import json

import os

from tqdm import tqdm

class PowerServiceDataset(Dataset):

"""Dataset class for power service process data."""

def __init__(self, data_path, mode='train'):

self.data = self._load_data(data_path, mode)

self.features = self.data['features']

self.labels = self.data['labels']

self.edge_index = self.data['edge_index']

def _load_data(self, data_path, mode):

# Load preprocessed data

data = np.load(os.path.join(data_path, f'{mode}_data.npz'), allow_pickle=True)

return {

'features': torch.FloatTensor(data['features']),

'labels': torch.LongTensor(data['labels']),

'edge_index': torch.LongTensor(data['edge_index'])

}

def __len__(self):

return len(self.labels)

def __getitem__(self, idx):

return self.features[idx], self.labels[idx]

def get_edge_index(self):

return self.edge_index

class EarlyStopping:

"""Early stopping handler."""

def __init__(self, patience=20, min_delta=1e-4):

self.patience = patience

self.min_delta = min_delta

self.counter = 0

self.best_score = None

self.early_stop = False

self.best_model = None

def __call__(self, score, model):

if self.best_score is None:

self.best_score = score

self.best_model = model.state_dict().copy()

elif score < self.best_score + self.min_delta:

self.counter += 1

if self.counter >= self.patience:

self.early_stop = True

else:

self.best_score = score

self.best_model = model.state_dict().copy()

self.counter = 0

def train_epoch(model, dataloader, optimizer, criterion, edge_index, device):

"""Train for one epoch."""

model.train()

total_loss = 0

all_preds = []

all_labels = []

for features, labels in tqdm(dataloader, desc='Training'):

features = features.to(device)

labels = labels.to(device)

edge_index_batch = edge_index.to(device)

optimizer.zero_grad()

logits, reconstruction, anomaly_scores = model(features, edge_index_batch)

loss, cls_loss, recon_loss = criterion(logits, labels, reconstruction, features)

loss.backward()

torch.nn.utils.clip_grad_norm_(model.parameters(), max_norm=5.0)

optimizer.step()

total_loss += loss.item()

preds = torch.argmax(logits, dim=1).cpu().numpy()

all_preds.extend(preds)

all_labels.extend(labels.cpu().numpy())

avg_loss = total_loss / len(dataloader)

accuracy = accuracy_score(all_labels, all_preds)

return avg_loss, accuracy

def evaluate(model, dataloader, criterion, edge_index, device):

"""Evaluate the model."""

model.eval()

total_loss = 0

all_preds = []

all_labels = []

all_probs = []

with torch.no_grad():

for features, labels in tqdm(dataloader, desc='Evaluating'):

features = features.to(device)

labels = labels.to(device)

edge_index_batch = edge_index.to(device)

logits, reconstruction, anomaly_scores = model(features, edge_index_batch)

loss, _, _ = criterion(logits, labels, reconstruction, features)

total_loss += loss.item()

probs = F.softmax(logits, dim=1)[:, 1].cpu().numpy()

preds = torch.argmax(logits, dim=1).cpu().numpy()

all_preds.extend(preds)

all_labels.extend(labels.cpu().numpy())

all_probs.extend(probs)

avg_loss = total_loss / len(dataloader)

metrics = {

'loss': avg_loss,

'accuracy': accuracy_score(all_labels, all_preds),

'precision': precision_score(all_labels, all_preds, zero_division=0),

'recall': recall_score(all_labels, all_preds, zero_division=0),

'f1': f1_score(all_labels, all_preds, zero_division=0),

'auc_roc': roc_auc_score(all_labels, all_probs) if len(set(all_labels)) > 1 else 0.5

}

return metrics

def train_model(config):

"""Main training function."""

device = torch.device('cuda' if torch.cuda.is_available() else 'cpu')

print(f'Using device: {device}')

# Load datasets

train_dataset = PowerServiceDataset(config['data_path'], mode='train')

val_dataset = PowerServiceDataset(config['data_path'], mode='val')

test_dataset = PowerServiceDataset(config['data_path'], mode='test')

train_loader = DataLoader(train_dataset, batch_size=config['batch_size'], shuffle=True)

val_loader = DataLoader(val_dataset, batch_size=config['batch_size'], shuffle=False)

test_loader = DataLoader(test_dataset, batch_size=config['batch_size'], shuffle=False)

edge_index = train_dataset.get_edge_index()

# Initialize model

model = SpatiotemporalAttentionModel(

input_dim=config['input_dim'],

d_model=config['d_model'],

n_heads=config['n_heads'],

n_temporal_layers=config['n_temporal_layers'],

n_spatial_layers=config['n_spatial_layers'],

d_ff=config['d_ff'],

max_seq_len=config['max_seq_len'],

n_nodes=config['n_nodes'],

n_classes=config['n_classes'],

dropout=config['dropout']

).to(device)

# Loss function with class weights

class_weights = torch.FloatTensor([1.0, config['anomaly_weight']]).to(device)

criterion = CombinedLoss(class_weights, gamma=config['focal_gamma'], recon_weight=config['recon_weight'])

# Optimizer and scheduler

optimizer = optim.AdamW(model.parameters(), lr=config['learning_rate'], weight_decay=config['weight_decay'])

scheduler = optim.lr_scheduler.ExponentialLR(optimizer, gamma=config['lr_decay'])

# Early stopping

early_stopping = EarlyStopping(patience=config['patience'])

# Training loop

history = {'train_loss': [], 'val_loss': [], 'val_metrics': []}

for epoch in range(config['max_epochs']):

print(f'\nEpoch {epoch + 1}/{config["max_epochs"]}')

# Train

train_loss, train_acc = train_epoch(model, train_loader, optimizer, criterion, edge_index, device)

print(f'Train Loss: {train_loss:.4f}, Train Acc: {train_acc:.4f}')

# Validate

val_metrics = evaluate(model, val_loader, criterion, edge_index, device)

print(f'Val Loss: {val_metrics["loss"]:.4f}, Val F1: {val_metrics["f1"]:.4f}')

history['train_loss'].append(train_loss)

history['val_loss'].append(val_metrics['loss'])

history['val_metrics'].append(val_metrics)

# Learning rate scheduling

if (epoch + 1) % config['lr_decay_step'] == 0:

scheduler.step()

# Early stopping check

early_stopping(val_metrics['f1'], model)

if early_stopping.early_stop:

print(f'Early stopping triggered at epoch {epoch + 1}')

break

# Load best model

model.load_state_dict(early_stopping.best_model)

# Test evaluation

test_metrics = evaluate(model, test_loader, criterion, edge_index, device)

print('\nTest Results:')

for key, value in test_metrics.items():

print(f' {key}: {value:.4f}')

# Save model and results

torch.save(model.state_dict(), os.path.join(config['output_dir'], 'best_model.pt'))

with open(os.path.join(config['output_dir'], 'training_history.json'), 'w') as f:

json.dump(history, f, indent=2)

with open(os.path.join(config['output_dir'], 'test_metrics.json'), 'w') as f:

json.dump(test_metrics, f, indent=2)

return model, test_metrics

# Configuration

config = {

'data_path': './data/processed/',

'output_dir': './outputs/',

'input_dim': 23,

'd_model': 128,

'n_heads': 8,

'n_temporal_layers': 4,

'n_spatial_layers': 3,

'd_ff': 512,

'max_seq_len': 50,

'n_nodes': 200,

'n_classes': 2,

'dropout': 0.2,

'batch_size': 64,

'learning_rate': 0.001,

'weight_decay': 1e-5,

'lr_decay': 0.95,

'lr_decay_step': 10,

'max_epochs': 200,

'patience': 20,

'anomaly_weight': 20.5,

'focal_gamma': 2.0,

'recon_weight': 0.1

}

if __name__ == '__main__':

os.makedirs(config['output_dir'], exist_ok=True)

model, metrics = train_model(config)

**S3.3 Baseline Model Implementations**

# Baseline implementations

import torch

import torch.nn as nn

from torch_geometric.nn import GCNConv

class LSTMBaseline(nn.Module):

"""LSTM baseline for anomaly detection."""

def __init__(self, input_dim, hidden_dim=128, num_layers=2, dropout=0.3, bidirectional=True):

super(LSTMBaseline, self).__init__()

self.lstm = nn.LSTM(

input_dim, hidden_dim, num_layers,

batch_first=True, dropout=dropout, bidirectional=bidirectional

)

fc_dim = hidden_dim * 2 if bidirectional else hidden_dim

self.classifier = nn.Sequential(

nn.Linear(fc_dim, hidden_dim),

nn.ReLU(),

nn.Dropout(dropout),

nn.Linear(hidden_dim, 2)

)

def forward(self, x):

# x: (batch, seq_len, n_nodes, features) -> flatten spatial

batch, seq_len, n_nodes, features = x.size()

x = x.view(batch, seq_len, n_nodes * features)

lstm_out, _ = self.lstm(x)

out = lstm_out[:, -1, :]

return self.classifier(out)

class GCNBaseline(nn.Module):

"""GCN baseline for anomaly detection."""

def __init__(self, input_dim, hidden_dim=128, num_layers=3, dropout=0.3):

super(GCNBaseline, self).__init__()

self.convs = nn.ModuleList()

self.convs.append(GCNConv(input_dim, hidden_dim))

for _ in range(num_layers - 1):

self.convs.append(GCNConv(hidden_dim, hidden_dim))

self.classifier = nn.Sequential(

nn.Linear(hidden_dim, hidden_dim),

nn.ReLU(),

nn.Dropout(dropout),

nn.Linear(hidden_dim, 2)

)

self.dropout = nn.Dropout(dropout)

def forward(self, x, edge_index):

# x: (batch * n_nodes, features)

for conv in self.convs:

x = conv(x, edge_index)

x = F.relu(x)

x = self.dropout(x)

return self.classifier(x)

class TransformerBaseline(nn.Module):

"""Transformer baseline for anomaly detection."""

def __init__(self, input_dim, d_model=128, nhead=8, num_layers=4, dim_feedforward=512, dropout=0.2):

super(TransformerBaseline, self).__init__()

self.embedding = nn.Linear(input_dim, d_model)

encoder_layer = nn.TransformerEncoderLayer(d_model, nhead, dim_feedforward, dropout, batch_first=True)

self.transformer = nn.TransformerEncoder(encoder_layer, num_layers)

self.classifier = nn.Sequential(

nn.Linear(d_model, dim_feedforward),

nn.ReLU(),

nn.Dropout(dropout),

nn.Linear(dim_feedforward, 2)

)

def forward(self, x):

# x: (batch, seq_len, n_nodes, features) -> flatten spatial

batch, seq_len, n_nodes, features = x.size()

x = x.view(batch, seq_len, n_nodes * features)

x = self.embedding(x)

x = self.transformer(x)

x = x.mean(dim=1)

return self.classifier(x)

class DCRNNCell(nn.Module):

"""Diffusion Convolutional GRU Cell."""

def __init__(self, input_dim, hidden_dim, num_nodes, K=2):

super(DCRNNCell, self).__init__()

self.hidden_dim = hidden_dim

self.K = K

self.gate = nn.Linear((K + 1) * (input_dim + hidden_dim), 2 * hidden_dim)

self.update = nn.Linear((K + 1) * (input_dim + hidden_dim), hidden_dim)

def forward(self, x, h, supports):

# Diffusion convolution

combined = torch.cat([x, h], dim=-1)

diffused = [combined]

for support in supports:

for k in range(self.K):

diffused.append(torch.matmul(support, diffused[-1] if k == 0 else diffused[-1]))

combined = torch.cat(diffused, dim=-1)

gates = torch.sigmoid(self.gate(combined))

r, u = gates.chunk(2, dim=-1)

combined_r = torch.cat([x, r * h], dim=-1)

diffused_r = [combined_r]

for support in supports:

for k in range(self.K):

diffused_r.append(torch.matmul(support, diffused_r[-1] if k == 0 else diffused_r[-1]))

combined_r = torch.cat(diffused_r, dim=-1)

c = torch.tanh(self.update(combined_r))

h_new = u * h + (1 - u) * c

return h_new

class DCRNNBaseline(nn.Module):

"""DCRNN baseline implementation."""

def __init__(self, input_dim, hidden_dim=64, num_layers=2, num_nodes=200, K=2):

super(DCRNNBaseline, self).__init__()

self.num_layers = num_layers

self.hidden_dim = hidden_dim

self.num_nodes = num_nodes

self.cells = nn.ModuleList([

DCRNNCell(input_dim if i == 0 else hidden_dim, hidden_dim, num_nodes, K)

for i in range(num_layers)

])

self.classifier = nn.Sequential(

nn.Linear(hidden_dim, hidden_dim),

nn.ReLU(),

nn.Linear(hidden_dim, 2)

)

def forward(self, x, supports):

batch, seq_len, n_nodes, features = x.size()

h = [torch.zeros(batch, n_nodes, self.hidden_dim, device=x.device) for _ in range(self.num_layers)]

for t in range(seq_len):

x_t = x[:, t, :, :]

for i, cell in enumerate(self.cells):

h[i] = cell(x_t if i == 0 else h[i-1], h[i], supports)

out = h[-1].mean(dim=1)

return self.classifier(out)

class ASTGCNBlock(nn.Module):

"""Spatial-Temporal Attention Block for ASTGCN."""

def __init__(self, in_channels, out_channels, num_nodes, K=3):

super(ASTGCNBlock, self).__init__()

self.spatial_attn = nn.MultiheadAttention(in_channels, num_heads=4, batch_first=True)

self.temporal_attn = nn.MultiheadAttention(in_channels, num_heads=4, batch_first=True)

self.cheb_conv = nn.Linear(in_channels * (K + 1), out_channels)

self.time_conv = nn.Conv1d(in_channels, out_channels, kernel_size=3, padding=1)

self.layer_norm = nn.LayerNorm(out_channels)

def forward(self, x):

batch, seq_len, n_nodes, features = x.size()

# Spatial attention

x_spatial = x.view(batch * seq_len, n_nodes, features)

x_spatial, _ = self.spatial_attn(x_spatial, x_spatial, x_spatial)

x_spatial = x_spatial.view(batch, seq_len, n_nodes, features)

# Temporal attention

x_temporal = x.permute(0, 2, 1, 3).contiguous().view(batch * n_nodes, seq_len, features)

x_temporal, _ = self.temporal_attn(x_temporal, x_temporal, x_temporal)

x_temporal = x_temporal.view(batch, n_nodes, seq_len, features).permute(0, 2, 1, 3)

# Combine

x = x_spatial + x_temporal

return self.layer_norm(x)

class ASTGCNBaseline(nn.Module):

"""ASTGCN baseline implementation."""

def __init__(self, input_dim, hidden_dim=64, num_blocks=3, num_nodes=200, K=3):

super(ASTGCNBaseline, self).__init__()

self.embedding = nn.Linear(input_dim, hidden_dim)

self.blocks = nn.ModuleList([

ASTGCNBlock(hidden_dim, hidden_dim, num_nodes, K)

for _ in range(num_blocks)

])

self.classifier = nn.Sequential(

nn.Linear(hidden_dim, hidden_dim),

nn.ReLU(),

nn.Linear(hidden_dim, 2)

)

def forward(self, x):

x = self.embedding(x)

for block in self.blocks:

x = block(x)

x = x.mean(dim=[1, 2])

return self.classifier(x)

class ProbSparseAttention(nn.Module):

"""ProbSparse self-attention for Informer."""

def __init__(self, d_model, n_heads, factor=5):

super(ProbSparseAttention, self).__init__()

self.d_model = d_model

self.n_heads = n_heads

self.d_k = d_model // n_heads

self.factor = factor

self.W_Q = nn.Linear(d_model, d_model)

self.W_K = nn.Linear(d_model, d_model)

self.W_V = nn.Linear(d_model, d_model)

self.W_O = nn.Linear(d_model, d_model)

def forward(self, x):

batch, seq_len, _ = x.size()

Q = self.W_Q(x).view(batch, seq_len, self.n_heads, self.d_k).transpose(1, 2)

K = self.W_K(x).view(batch, seq_len, self.n_heads, self.d_k).transpose(1, 2)

V = self.W_V(x).view(batch, seq_len, self.n_heads, self.d_k).transpose(1, 2)

# Sample top-k queries based on sparsity measurement

u = self.factor * int(np.ceil(np.log(seq_len)))

u = min(u, seq_len)

scores = torch.matmul(Q, K.transpose(-2, -1)) / np.sqrt(self.d_k)

attn = F.softmax(scores, dim=-1)

context = torch.matmul(attn, V)

context = context.transpose(1, 2).contiguous().view(batch, seq_len, self.d_model)

return self.W_O(context)

class InformerBaseline(nn.Module):

"""Informer baseline implementation."""

def __init__(self, input_dim, d_model=128, n_heads=8, e_layers=3, d_layers=2, factor=5, dropout=0.2):

super(InformerBaseline, self).__init__()

self.embedding = nn.Linear(input_dim, d_model)

self.encoder_layers = nn.ModuleList([

nn.Sequential(

ProbSparseAttention(d_model, n_heads, factor),

nn.LayerNorm(d_model),

nn.Linear(d_model, d_model * 4),

nn.GELU(),

nn.Linear(d_model * 4, d_model),

nn.Dropout(dropout)

) for _ in range(e_layers)

])

self.classifier = nn.Sequential(

nn.Linear(d_model, d_model),

nn.ReLU(),

nn.Dropout(dropout),

nn.Linear(d_model, 2)

)

def forward(self, x):

batch, seq_len, n_nodes, features = x.size()

x = x.view(batch, seq_len, n_nodes * features)

x = self.embedding(x)

for layer in self.encoder_layers:

x = x + layer(x)

x = x.mean(dim=1)

return self.classifier(x)

class Inception_Block_V1(nn.Module):

"""Inception block for TimesNet."""

def __init__(self, in_channels, out_channels, num_kernels=6):

super(Inception_Block_V1, self).__init__()

self.convs = nn.ModuleList([

nn.Conv2d(in_channels, out_channels, kernel_size=(1, 2*i+1), padding=(0, i))

for i in range(num_kernels)

])

def forward(self, x):

out = sum([conv(x) for conv in self.convs])

return out

class TimesBlock(nn.Module):

"""TimesNet block."""

def __init__(self, d_model, d_ff, top_k=3, num_kernels=6):

super(TimesBlock, self).__init__()

self.top_k = top_k

self.conv = nn.Sequential(

Inception_Block_V1(d_model, d_ff, num_kernels),

nn.GELU(),

Inception_Block_V1(d_ff, d_model, num_kernels)

)

def forward(self, x):

batch, seq_len, d_model = x.size()

# FFT to find top-k periods

xf = torch.fft.rfft(x, dim=1)

freq = torch.abs(xf).mean(dim=-1)

_, top_idx = torch.topk(freq, self.top_k, dim=1)

# Process each period

period_weights = torch.softmax(freq.gather(1, top_idx), dim=1)

# 2D representation

res = []

for i in range(self.top_k):

period = max(seq_len // (top_idx[:, i].float().mean().item() + 1), 2)

period = int(period)

if seq_len % period != 0:

pad_len = period - (seq_len % period)

x_pad = F.pad(x, (0, 0, 0, pad_len))

else:

x_pad = x

pad_len = 0

x_2d = x_pad.view(batch, -1, period, d_model).permute(0, 3, 1, 2)

x_2d = self.conv(x_2d)

x_2d = x_2d.permute(0, 2, 3, 1).contiguous().view(batch, -1, d_model)

if pad_len > 0:

x_2d = x_2d[:, :seq_len, :]

res.append(x_2d)

res = torch.stack(res, dim=-1)

res = (res * period_weights.unsqueeze(1).unsqueeze(1)).sum(dim=-1)

return x + res

class TimesNetBaseline(nn.Module):

"""TimesNet baseline implementation."""

def __init__(self, input_dim, d_model=128, d_ff=256, num_layers=2, top_k=3, num_kernels=6, dropout=0.2):

super(TimesNetBaseline, self).__init__()

self.embedding = nn.Linear(input_dim, d_model)

self.blocks = nn.ModuleList([

TimesBlock(d_model, d_ff, top_k, num_kernels)

for _ in range(num_layers)

])

self.layer_norm = nn.LayerNorm(d_model)

self.classifier = nn.Sequential(

nn.Linear(d_model, d_ff),

nn.ReLU(),

nn.Dropout(dropout),

nn.Linear(d_ff, 2)

)

def forward(self, x):

batch, seq_len, n_nodes, features = x.size()

x = x.view(batch, seq_len, n_nodes * features)

x = self.embedding(x)

for block in self.blocks:

x = block(x)

x = self.layer_norm(x)

x = x.mean(dim=1)

return self.classifier(x)

**S4. Evaluation Metrics Implementation**

import numpy as np

from sklearn.metrics import (

accuracy_score, precision_score, recall_score, f1_score,

roc_auc_score, precision_recall_curve, auc, confusion_matrix,

classification_report

)

from scipy import stats

import torch

def compute_metrics(y_true, y_pred, y_prob=None):

"""Compute comprehensive evaluation metrics."""

metrics = {

'accuracy': accuracy_score(y_true, y_pred),

'precision': precision_score(y_true, y_pred, zero_division=0),

'recall': recall_score(y_true, y_pred, zero_division=0),

'f1_score': f1_score(y_true, y_pred, zero_division=0),

'specificity': None,

'auc_roc': None,

'auc_pr': None

}

# Confusion matrix derived metrics

cm = confusion_matrix(y_true, y_pred)

if cm.shape == (2, 2):

tn, fp, fn, tp = cm.ravel()

metrics['specificity'] = tn / (tn + fp) if (tn + fp) > 0 else 0

metrics['true_positives'] = int(tp)

metrics['true_negatives'] = int(tn)

metrics['false_positives'] = int(fp)

metrics['false_negatives'] = int(fn)

# Probability-based metrics

if y_prob is not None:

try:

metrics['auc_roc'] = roc_auc_score(y_true, y_prob)

precision_curve, recall_curve, _ = precision_recall_curve(y_true, y_prob)

metrics['auc_pr'] = auc(recall_curve, precision_curve)

except ValueError:

metrics['auc_roc'] = 0.5

metrics['auc_pr'] = 0.5

return metrics

def compute_per_class_metrics(y_true, y_pred, class_names=None):

"""Compute per-class metrics."""

if class_names is None:

class_names = ['Normal', 'Anomaly']

report = classification_report(y_true, y_pred, target_names=class_names, output_dict=True)

return report

def compute_confidence_interval(data, confidence=0.95):

"""Compute confidence interval using bootstrap."""

n = len(data)

mean = np.mean(data)

se = stats.sem(data)

h = se * stats.t.ppf((1 + confidence) / 2, n - 1)

return mean, mean - h, mean + h

def bootstrap_metrics(y_true, y_pred, y_prob=None, n_iterations=10000, confidence=0.95):

"""Compute metrics with bootstrap confidence intervals."""

n = len(y_true)

metrics_list = {

'accuracy': [], 'precision': [], 'recall': [], 'f1_score': [], 'auc_roc': []

}

for _ in range(n_iterations):

indices = np.random.choice(n, n, replace=True)

y_true_sample = np.array(y_true)[indices]

y_pred_sample = np.array(y_pred)[indices]

if len(np.unique(y_true_sample)) < 2:

continue

metrics_list['accuracy'].append(accuracy_score(y_true_sample, y_pred_sample))

metrics_list['precision'].append(precision_score(y_true_sample, y_pred_sample, zero_division=0))

metrics_list['recall'].append(recall_score(y_true_sample, y_pred_sample, zero_division=0))

metrics_list['f1_score'].append(f1_score(y_true_sample, y_pred_sample, zero_division=0))

if y_prob is not None:

y_prob_sample = np.array(y_prob)[indices]

try:

metrics_list['auc_roc'].append(roc_auc_score(y_true_sample, y_prob_sample))

except ValueError:

pass

results = {}

for metric, values in metrics_list.items():

if len(values) > 0:

mean, lower, upper = compute_confidence_interval(values, confidence)

results[metric] = {

'mean': mean,

'std': np.std(values),

'ci_lower': lower,

'ci_upper': upper

}

return results

def delong_test(y_true, y_prob1, y_prob2):

"""DeLong test for comparing two AUC-ROC values."""

from scipy.stats import norm

n1 = sum(y_true == 1)

n0 = sum(y_true == 0)

y_true = np.array(y_true)

y_prob1 = np.array(y_prob1)

y_prob2 = np.array(y_prob2)

# Compute AUCs

auc1 = roc_auc_score(y_true, y_prob1)

auc2 = roc_auc_score(y_true, y_prob2)

# Structural components

pos_idx = y_true == 1

neg_idx = y_true == 0

# Placement values

V10_1 = np.array([np.mean(y_prob1[neg_idx] < p) + 0.5 * np.mean(y_prob1[neg_idx] == p) for p in y_prob1[pos_idx]])

V01_1 = np.array([np.mean(y_prob1[pos_idx] > p) + 0.5 * np.mean(y_prob1[pos_idx] == p) for p in y_prob1[neg_idx]])

V10_2 = np.array([np.mean(y_prob2[neg_idx] < p) + 0.5 * np.mean(y_prob2[neg_idx] == p) for p in y_prob2[pos_idx]])

V01_2 = np.array([np.mean(y_prob2[pos_idx] > p) + 0.5 * np.mean(y_prob2[pos_idx] == p) for p in y_prob2[neg_idx]])

# Variance

S10 = np.cov(V10_1, V10_2)

S01 = np.cov(V01_1, V01_2)

S = S10 / n1 + S01 / n0

# Test statistic

diff = auc1 - auc2

var = S[0, 0] + S[1, 1] - 2 * S[0, 1]

if var <= 0:

return diff, 1.0

z = diff / np.sqrt(var)

p_value = 2 * (1 - norm.cdf(abs(z)))

return diff, p_value

def compute_anomaly_type_metrics(y_true, y_pred, anomaly_types):

"""Compute metrics per anomaly type."""

unique_types = np.unique(anomaly_types)

results = {}

for atype in unique_types:

if atype == 'none':

continue

mask = anomaly_types == atype

if mask.sum() == 0:

continue

y_true_type = y_true[mask]

y_pred_type = y_pred[mask]

results[atype] = {

'count': int(mask.sum()),

'precision': precision_score(y_true_type, y_pred_type, zero_division=0),

'recall': recall_score(y_true_type, y_pred_type, zero_division=0),

'f1_score': f1_score(y_true_type, y_pred_type, zero_division=0)

}

return results

**S5. Attention Visualization Tools**

import matplotlib.pyplot as plt

import seaborn as sns

import numpy as np

import torch

def visualize_temporal_attention(attention_weights, time_labels=None, save_path=None):

"""Visualize temporal attention weights."""

plt.figure(figsize=(12, 6))

if isinstance(attention_weights, torch.Tensor):

attention_weights = attention_weights.detach().cpu().numpy()

# Average across heads if multi-head

if len(attention_weights.shape) > 2:

attention_weights = attention_weights.mean(axis=0)

sns.heatmap(attention_weights, cmap='YlOrRd', xticklabels=time_labels, yticklabels=time_labels)

plt.xlabel('Key Time Steps')

plt.ylabel('Query Time Steps')

plt.title('Temporal Attention Weights')

if save_path:

plt.savefig(save_path, dpi=300, bbox_inches='tight')

plt.close()

def visualize_spatial_attention(attention_weights, node_labels=None, adjacency_matrix=None, save_path=None):

"""Visualize spatial attention weights."""

plt.figure(figsize=(10, 10))

if isinstance(attention_weights, torch.Tensor):

attention_weights = attention_weights.detach().cpu().numpy()

# Average across heads if multi-head

if len(attention_weights.shape) > 2:

attention_weights = attention_weights.mean(axis=0)

sns.heatmap(attention_weights, cmap='Blues',

xticklabels=node_labels if len(node_labels) < 50 else False,

yticklabels=node_labels if len(node_labels) < 50 else False)

plt.xlabel('Target Nodes')

plt.ylabel('Source Nodes')

plt.title('Spatial Attention Weights')

if save_path:

plt.savefig(save_path, dpi=300, bbox_inches='tight')

plt.close()

def visualize_attention_over_process(model, sample, edge_index, stage_names, save_path=None):

"""Visualize attention weights over process stages."""

model.eval()

with torch.no_grad():

_, _, _, attention_info = model(sample.unsqueeze(0), edge_index, return_attention=True)

temporal_weights = attention_info['temporal_weights'][-1] # Last layer

if isinstance(temporal_weights, torch.Tensor):

temporal_weights = temporal_weights.detach().cpu().numpy()

# Average across batch and heads

weights = temporal_weights.mean(axis=(0, 1))

fig, ax = plt.subplots(figsize=(14, 6))

x = np.arange(len(stage_names))

bars = ax.bar(x, weights[:len(stage_names)], color='steelblue', alpha=0.8)

# Highlight high-attention stages

threshold = np.percentile(weights, 75)

for i, (bar, w) in enumerate(zip(bars, weights)):

if w > threshold:

bar.set_color('crimson')

ax.set_xticks(x)

ax.set_xticklabels(stage_names, rotation=45, ha='right')

ax.set_ylabel('Attention Weight')

ax.set_title('Temporal Attention Distribution Across Process Stages')

plt.tight_layout()

if save_path:

plt.savefig(save_path, dpi=300, bbox_inches='tight')

plt.close()

def visualize_gate_distribution(gates, save_path=None):

"""Visualize gating mechanism distribution."""

if isinstance(gates, torch.Tensor):

gates = gates.detach().cpu().numpy()

# Flatten if needed

gates = gates.flatten()

fig, axes = plt.subplots(1, 2, figsize=(12, 5))

# Histogram

axes[0].hist(gates, bins=50, color='teal', alpha=0.7, edgecolor='black')

axes[0].axvline(x=0.5, color='red', linestyle='--', label='Balance point')

axes[0].set_xlabel('Gate Value')

axes[0].set_ylabel('Frequency')

axes[0].set_title('Distribution of Fusion Gate Values')

axes[0].legend()

# Time evolution (if 2D)

axes[1].plot(gates[:100], color='teal', linewidth=1.5)

axes[1].axhline(y=0.5, color='red', linestyle='--', alpha=0.5)

axes[1].fill_between(range(100), 0, gates[:100], alpha=0.3)

axes[1].set_xlabel('Sample Index')

axes[1].set_ylabel('Gate Value')

axes[1].set_title('Gate Values Over Samples')

axes[1].set_ylim(0, 1)

plt.tight_layout()

if save_path:

plt.savefig(save_path, dpi=300, bbox_inches='tight')

plt.close()

def visualize_anomaly_attribution(model, sample, edge_index, feature_names, save_path=None):

"""Visualize feature attribution for anomaly detection."""

model.eval()

sample.requires_grad_(True)

logits, _, anomaly_scores = model(sample.unsqueeze(0), edge_index)

# Compute gradients

anomaly_score = anomaly_scores['composite']

anomaly_score.backward()

# Get attribution

attribution = sample.grad.abs().mean(dim=(0, 1, 2)).detach().cpu().numpy()

# Normalize

attribution = attribution / attribution.sum()

# Plot

fig, ax = plt.subplots(figsize=(12, 6))

y_pos = np.arange(len(feature_names))

bars = ax.barh(y_pos, attribution, color='coral', alpha=0.8)

# Highlight top features

top_k = 5

top_indices = np.argsort(attribution)[-top_k:]

for idx in top_indices:

bars[idx].set_color('crimson')

ax.set_yticks(y_pos)

ax.set_yticklabels(feature_names)

ax.set_xlabel('Attribution Score')

ax.set_title('Feature Attribution for Anomaly Detection')

plt.tight_layout()

if save_path:

plt.savefig(save_path, dpi=300, bbox_inches='tight')

plt.close()

def create_attention_report(model, test_loader, edge_index, output_dir):

"""Generate comprehensive attention analysis report."""

import os

os.makedirs(output_dir, exist_ok=True)

model.eval()

all_temporal_weights = []

all_spatial_weights = []

all_gates = []

with torch.no_grad():

for batch_idx, (features, labels) in enumerate(test_loader):

if batch_idx >= 10: # Limit samples for visualization

break

_, _, _, attention_info = model(features, edge_index, return_attention=True)

all_temporal_weights.append(attention_info['temporal_weights'][-1])

all_gates.append(attention_info['gates'][-1])

# Aggregate

temporal_weights = torch.cat(all_temporal_weights, dim=0).mean(dim=0)

gates = torch.cat(all_gates, dim=0)

# Generate visualizations

visualize_temporal_attention(

temporal_weights,

save_path=os.path.join(output_dir, 'temporal_attention.png')

)

visualize_gate_distribution(

gates,

save_path=os.path.join(output_dir, 'gate_distribution.png')

)

print(f'Attention analysis report saved to {output_dir}')

**S6. Data Preprocessing Pipeline**

import pandas as pd

import numpy as np

from sklearn.preprocessing import StandardScaler, LabelEncoder

from datetime import datetime

import torch

class PowerServiceDataPreprocessor:

"""Preprocessing pipeline for power service data."""

def __init__(self, config):

self.config = config

self.scalers = {}

self.encoders = {}

self.feature_columns = None

def fit_transform(self, df):

"""Fit preprocessors and transform data."""

# Handle missing values

df = self._handle_missing(df)

# Extract temporal features

df = self._extract_temporal_features(df)

# Encode categorical features

df = self._encode_categorical(df, fit=True)

# Scale numerical features

df = self._scale_numerical(df, fit=True)

# Create spatiotemporal tensors

tensors = self._create_tensors(df)

return tensors

def transform(self, df):

"""Transform data using fitted preprocessors."""

df = self._handle_missing(df)

df = self._extract_temporal_features(df)

df = self._encode_categorical(df, fit=False)

df = self._scale_numerical(df, fit=False)

tensors = self._create_tensors(df)

return tensors

def _handle_missing(self, df):

"""Handle missing values."""

# Forward fill for temporal continuity

temporal_cols = ['stage_duration_hours', 'workload_index', 'capacity_utilization']

for col in temporal_cols:

if col in df.columns:

df[col] = df.groupby('process_id')[col].ffill()

# Fill remaining with median

numeric_cols = df.select_dtypes(include=[np.number]).columns

for col in numeric_cols:

if df[col].isna().any():

df[col] = df[col].fillna(df[col].median())

# Fill categorical with mode

cat_cols = df.select_dtypes(include=['object', 'category']).columns

for col in cat_cols:

if df[col].isna().any():

df[col] = df[col].fillna(df[col].mode()[0])

return df

def _extract_temporal_features(self, df):

"""Extract temporal features from timestamps."""

if 'stage_start_time' in df.columns:

df['stage_start_time'] = pd.to_datetime(df['stage_start_time'])

df['hour_of_day'] = df['stage_start_time'].dt.hour

df['day_of_week'] = df['stage_start_time'].dt.dayofweek

df['month'] = df['stage_start_time'].dt.month

df['is_weekend'] = (df['day_of_week'] >= 5).astype(int)

# Cyclic encoding

df['hour_sin'] = np.sin(2 * np.pi * df['hour_of_day'] / 24)

df['hour_cos'] = np.cos(2 * np.pi * df['hour_of_day'] / 24)

df['day_sin'] = np.sin(2 * np.pi * df['day_of_week'] / 7)

df['day_cos'] = np.cos(2 * np.pi * df['day_of_week'] / 7)

df['month_sin'] = np.sin(2 * np.pi * df['month'] / 12)

df['month_cos'] = np.cos(2 * np.pi * df['month'] / 12)

return df

def _encode_categorical(self, df, fit=True):

"""Encode categorical features."""

cat_cols = ['customer_type', 'application_type', 'stage_name']

for col in cat_cols:

if col not in df.columns:

continue

if fit:

self.encoders[col] = LabelEncoder()

df[f'{col}_encoded'] = self.encoders[col].fit_transform(df[col].astype(str))

else:

# Handle unseen categories

known_classes = set(self.encoders[col].classes_)

df[col] = df[col].apply(lambda x: x if x in known_classes else 'unknown')

if 'unknown' not in self.encoders[col].classes_:

self.encoders[col].classes_ = np.append(self.encoders[col].classes_, 'unknown')

df[f'{col}_encoded'] = self.encoders[col].transform(df[col].astype(str))

return df

def _scale_numerical(self, df, fit=True):

"""Scale numerical features."""

num_cols = [

'stage_duration_hours', 'workload_index', 'capacity_utilization',

'latitude', 'longitude', 'application_complexity', 'assigned_personnel'

]

existing_cols = [col for col in num_cols if col in df.columns]

if fit:

self.scalers['numerical'] = StandardScaler()

df[existing_cols] = self.scalers['numerical'].fit_transform(df[existing_cols])

else:

df[existing_cols] = self.scalers['numerical'].transform(df[existing_cols])

return df

def _create_tensors(self, df):

"""Create spatiotemporal tensors from processed data."""

# Define feature columns

feature_cols = [

'stage_duration_hours', 'workload_index', 'capacity_utilization',

'latitude', 'longitude', 'application_complexity', 'assigned_personnel',

'customer_type_encoded', 'application_type_encoded', 'stage_name_encoded',

'hour_sin', 'hour_cos', 'day_sin', 'day_cos', 'month_sin', 'month_cos',

'is_holiday', 'is_weekend', 'is_peak_season'

]

self.feature_columns = [col for col in feature_cols if col in df.columns]

# Group by process_id

grouped = df.groupby('process_id')

features_list = []

labels_list = []

max_seq_len = self.config.get('max_seq_len', 50)

n_nodes = self.config.get('n_nodes', 200)

for process_id, group in grouped:

# Get features

process_features = group[self.feature_columns].values

# Pad or truncate sequence

if len(process_features) < max_seq_len:

padding = np.zeros((max_seq_len - len(process_features), len(self.feature_columns)))

process_features = np.vstack([process_features, padding])

else:

process_features = process_features[:max_seq_len]

# Expand to spatial dimension (service center assignment)

center_id = group['service_center_id'].iloc[0] - 1 # 0-indexed

spatial_features = np.zeros((max_seq_len, n_nodes, len(self.feature_columns)))

spatial_features[:, center_id, :] = process_features

features_list.append(spatial_features)

labels_list.append(group['is_anomaly'].iloc[0])

features = np.array(features_list, dtype=np.float32)

labels = np.array(labels_list, dtype=np.int64)

return {

'features': torch.FloatTensor(features),

'labels': torch.LongTensor(labels)

}

def create_adjacency_matrix(self, center_info_df):

"""Create spatial adjacency matrix based on geographic proximity and administrative hierarchy."""

n_physical = len(center_info_df)

n_virtual = 44 # District and provincial aggregation nodes

n_total = n_physical + n_virtual

adj_matrix = np.zeros((n_total, n_total))

# Physical center connections

coords = center_info_df[['latitude', 'longitude']].values

for i in range(n_physical):

for j in range(i + 1, n_physical):

# Geographic distance

dist = np.sqrt(np.sum((coords[i] - coords[j]) ** 2)) * 111 # Approx km

# Same administrative district

same_district = center_info_df.iloc[i]['district_id'] == center_info_df.iloc[j]['district_id']

if dist < 50 or same_district:

adj_matrix[i, j] = 1

adj_matrix[j, i] = 1

# Virtual node connections (hierarchical)

for i in range(n_physical):

district_node = n_physical + center_info_df.iloc[i]['district_id']

province_node = n_physical + 32 + center_info_df.iloc[i]['province_id']

adj_matrix[i, district_node] = 1

adj_matrix[district_node, i] = 1

adj_matrix[district_node, province_node] = 1

adj_matrix[province_node, district_node] = 1

# Self-loops

np.fill_diagonal(adj_matrix, 1)

# Convert to edge index

edge_index = np.array(np.nonzero(adj_matrix))

return torch.LongTensor(edge_index)

def preprocess_and_save(raw_data_path, output_path, config):

"""Main preprocessing function."""

import os

# Load raw data

df = pd.read_csv(raw_data_path)

center_info = pd.read_csv(os.path.join(os.path.dirname(raw_data_path), 'center_info.csv'))

preprocessor = PowerServiceDataPreprocessor(config)

# Split by date

df['date'] = pd.to_datetime(df['stage_start_time']).dt.date

train_mask = df['date'] < pd.to_datetime('2023-10-01').date()

val_mask = (df['date'] >= pd.to_datetime('2023-10-01').date()) & (df['date'] < pd.to_datetime('2024-04-01').date())

test_mask = df['date'] >= pd.to_datetime('2024-04-01').date()

# Process splits

train_data = preprocessor.fit_transform(df[train_mask].copy())

val_data = preprocessor.transform(df[val_mask].copy())

test_data = preprocessor.transform(df[test_mask].copy())

# Create adjacency

edge_index = preprocessor.create_adjacency_matrix(center_info)

# Save

os.makedirs(output_path, exist_ok=True)

np.savez(os.path.join(output_path, 'train_data.npz'),

features=train_data['features'].numpy(),

labels=train_data['labels'].numpy(),

edge_index=edge_index.numpy())

np.savez(os.path.join(output_path, 'val_data.npz'),

features=val_data['features'].numpy(),

labels=val_data['labels'].numpy(),

edge_index=edge_index.numpy())

np.savez(os.path.join(output_path, 'test_data.npz'),

features=test_data['features'].numpy(),

labels=test_data['labels'].numpy(),

edge_index=edge_index.numpy())

print(f'Preprocessed data saved to {output_path}')

print(f'Train samples: {len(train_data["labels"])}')

print(f'Val samples: {len(val_data["labels"])}')

print(f'Test samples: {len(test_data["labels"])}')

**S7. Adaptive Threshold Computation**

import numpy as np

from scipy import stats

from collections import deque

class AdaptiveThresholdManager:

"""Adaptive threshold management for anomaly detection."""

def __init__(self, k_base=2.75, delta=0.15, window_size=4, update_frequency='weekly'):

self.k_base = k_base

self.delta = delta

self.window_size = window_size

self.update_frequency = update_frequency

# Regional thresholds: {center_id: {period: threshold}}

self.thresholds = {}

# Score history: {center_id: deque}

self.score_history = {}

# Seasonal indices: {month: index}

self.seasonal_indices = {

1: 0.85, 2: 0.72, 3: 0.88, 4: 0.95,

5: 1.02, 6: 1.18, 7: 1.35, 8: 1.38,

9: 1.12, 10: 1.05, 11: 1.22, 12: 1.28

}

def compute_threshold(self, center_id, period, scores=None):

"""Compute adaptive threshold for a service center and period."""

if scores is None:

scores = self._get_historical_scores(center_id, period)

if len(scores) < 10:

# Insufficient history, use global default

return self._get_global_threshold(period)

mu = np.mean(scores)

sigma = np.std(scores)

# Get seasonal adjustment

month = period.month if hasattr(period, 'month') else period

seasonal_index = self.seasonal_indices.get(month, 1.0)

# Compute adaptive k

k = self.k_base * (1 + self.delta * (seasonal_index - 1))

threshold = mu + k * sigma

# Store threshold

if center_id not in self.thresholds:

self.thresholds[center_id] = {}

self.thresholds[center_id][period] = threshold

return threshold

def _get_historical_scores(self, center_id, period):

"""Get historical anomaly scores for threshold computation."""

if center_id not in self.score_history:

return []

history = self.score_history[center_id]

return list(history)

def _get_global_threshold(self, period):

"""Get global default threshold when regional history is insufficient."""

month = period.month if hasattr(period, 'month') else period

seasonal_index = self.seasonal_indices.get(month, 1.0)

return 0.5 * (1 + self.delta * (seasonal_index - 1))

def update_history(self, center_id, score):

"""Update score history with new observation."""

if center_id not in self.score_history:

self.score_history[center_id] = deque(maxlen=self.window_size * 7) # ~4 weeks

self.score_history[center_id].append(score)

def detect_concept_drift(self, center_id, recent_scores, significance=0.05):

"""Detect concept drift using Kolmogorov-Smirnov test."""

if center_id not in self.score_history:

return False, 1.0

historical = np.array(list(self.score_history[center_id]))

recent = np.array(recent_scores)

if len(historical) < 20 or len(recent) < 10:

return False, 1.0

statistic, p_value = stats.ks_2samp(historical, recent)

drift_detected = p_value < significance

return drift_detected, p_value

def get_threshold(self, center_id, period):

"""Get threshold for detection."""

if center_id in self.thresholds and period in self.thresholds[center_id]:

return self.thresholds[center_id][period]

return self.compute_threshold(center_id, period)

def batch_update_thresholds(self, scores_df):

"""Batch update thresholds for all centers."""

updated_thresholds = {}

for center_id in scores_df['center_id'].unique():

center_scores = scores_df[scores_df['center_id'] == center_id]

for period in center_scores['period'].unique():

period_scores = center_scores[center_scores['period'] == period]['score'].values

threshold = self.compute_threshold(center_id, period, period_scores)

updated_thresholds[(center_id, period)] = threshold

return updated_thresholds

def evaluate_threshold_performance(self, scores, labels, center_id, period):

"""Evaluate threshold performance for a specific setting."""

threshold = self.get_threshold(center_id, period)

predictions = (scores > threshold).astype(int)

tp = np.sum((predictions == 1) & (labels == 1))

fp = np.sum((predictions == 1) & (labels == 0))

fn = np.sum((predictions == 0) & (labels == 1))

tn = np.sum((predictions == 0) & (labels == 0))

precision = tp / (tp + fp) if (tp + fp) > 0 else 0

recall = tp / (tp + fn) if (tp + fn) > 0 else 0

f1 = 2 * precision * recall / (precision + recall) if (precision + recall) > 0 else 0

return {

'threshold': threshold,

'precision': precision,

'recall': recall,

'f1': f1,

'tp': tp, 'fp': fp, 'fn': fn, 'tn': tn

}

class AlertSuppressionManager:

"""Manage alert suppression during high-volume periods."""

def __init__(self, temporal_window=4, spatial_correlation_threshold=0.8, confidence_threshold=0.85):

self.temporal_window = temporal_window # hours

self.spatial_correlation_threshold = spatial_correlation_threshold

self.confidence_threshold = confidence_threshold

self.alert_buffer = {} # {center_id: [(timestamp, alert_info), ...]}

self.suppressed_count = 0

self.total_alerts = 0

def process_alert(self, alert_info):

"""Process incoming alert with suppression logic."""

self.total_alerts += 1

center_id = alert_info['center_id']

timestamp = alert_info['timestamp']

confidence = alert_info['confidence']

# Check temporal aggregation

if self._should_aggregate_temporally(center_id, timestamp):

self.suppressed_count += 1

self._update_aggregated_alert(center_id, alert_info)

return None

# Check spatial clustering

if self._should_cluster_spatially(alert_info):

self.suppressed_count += 1

return self._create_regional_alert(alert_info)

# Check confidence threshold during peak

if self._is_peak_period(timestamp) and confidence < self.confidence_threshold:

self.suppressed_count += 1

self._queue_for_batch_review(alert_info)

return None

# Pass through alert

self._add_to_buffer(center_id, timestamp, alert_info)

return alert_info

def _should_aggregate_temporally(self, center_id, timestamp):

"""Check if alert should be aggregated with recent alerts."""

if center_id not in self.alert_buffer:

return False

recent_alerts = self.alert_buffer[center_id]

for prev_timestamp, _ in recent_alerts:

time_diff = (timestamp - prev_timestamp).total_seconds() / 3600

if time_diff < self.temporal_window:

return True

return False

def _should_cluster_spatially(self, alert_info):

"""Check if alert should be clustered with regional alerts."""

# Simplified: check if neighboring centers have recent alerts

neighboring_centers = alert_info.get('neighboring_centers', [])

correlated_count = 0

for neighbor_id in neighboring_centers:

if neighbor_id in self.alert_buffer and len(self.alert_buffer[neighbor_id]) > 0:

correlated_count += 1

correlation = correlated_count / len(neighboring_centers) if neighboring_centers else 0

return correlation >= self.spatial_correlation_threshold

def _is_peak_period(self, timestamp):

"""Check if current time is in peak period."""

hour = timestamp.hour

month = timestamp.month

# Peak hours: 9-11 AM, 2-5 PM

peak_hours = hour in [9, 10, 11, 14, 15, 16, 17]

# Peak months: July, August, November, December

peak_months = month in [7, 8, 11, 12]

return peak_hours and peak_months

def _add_to_buffer(self, center_id, timestamp, alert_info):

"""Add alert to buffer."""

if center_id not in self.alert_buffer:

self.alert_buffer[center_id] = deque(maxlen=100)

self.alert_buffer[center_id].append((timestamp, alert_info))

def _update_aggregated_alert(self, center_id, alert_info):

"""Update aggregated alert with new information."""

pass # Implementation depends on specific aggregation logic

def _create_regional_alert(self, alert_info):

"""Create regional summary alert."""

return {

'type': 'regional',

'affected_centers': [alert_info['center_id']] + alert_info.get('neighboring_centers', []),

'summary': f"Regional anomaly pattern detected",

'timestamp': alert_info['timestamp'],

'confidence': alert_info['confidence']

}

def _queue_for_batch_review(self, alert_info):

"""Queue low-confidence alert for batch review."""

pass # Implementation for batch review queue

def get_suppression_stats(self):

"""Get suppression statistics."""

return {

'total_alerts': self.total_alerts,

'suppressed_alerts': self.suppressed_count,

'suppression_rate': self.suppressed_count / self.total_alerts if self.total_alerts > 0 else 0,

'passed_alerts': self.total_alerts - self.suppressed_count

}

**S8. Sample Anonymized Data Format**

**Table S6. Sample Data Records (First 10 rows)**

| **process_id** | **customer_type** | **application_type** | **service_center_id** | **region_id** | **stage_id** | **stage_duration_hours** | **workload_index** | **is_anomaly** | **anomaly_type** |
| --- | --- | --- | --- | --- | --- | --- | --- | --- | --- |
| PROC_00001 | residential | new_connection | 23 | 3 | 1 | 2.5 | 0.72 | 0 | none |
| PROC_00001 | residential | new_connection | 23 | 3 | 2 | 4.8 | 0.74 | 0 | none |
| PROC_00001 | residential | new_connection | 23 | 3 | 3 | 18.2 | 0.71 | 0 | none |
| PROC_00002 | commercial | capacity_upgrade | 45 | 5 | 1 | 3.1 | 0.85 | 0 | none |
| PROC_00002 | commercial | capacity_upgrade | 45 | 5 | 2 | 6.2 | 0.88 | 0 | none |
| PROC_00002 | commercial | capacity_upgrade | 45 | 5 | 4 | 156.3 | 0.92 | 1 | duration |
| PROC_00003 | industrial | new_connection | 78 | 8 | 1 | 4.2 | 0.65 | 0 | none |
| PROC_00003 | industrial | new_connection | 78 | 8 | 3 | 22.1 | 0.68 | 0 | none |
| PROC_00003 | industrial | new_connection | 78 | 8 | 6 | 0.0 | 0.71 | 1 | sequence |
| PROC_00004 | residential | relocation | 12 | 2 | 1 | 1.8 | 1.45 | 1 | resource |

**S9. Requirements and Environment Setup**

**requirements.txt**

torch==2.1.0

torch-geometric==2.4.0

numpy==1.24.3

pandas==1.5.3

scikit-learn==1.3.0

scipy==1.11.3

matplotlib==3.7.1

seaborn==0.12.2

tqdm==4.66.1

tensorboard==2.14.0

**environment.yml**

name: power_service_monitoring

channels:

- pytorch

- pyg

- conda-forge

- defaults

dependencies:

- python=3.9.18

- pytorch=2.1.0

- pytorch-cuda=12.1

- pyg=2.4.0

- numpy=1.24.3

- pandas=1.5.3

- scikit-learn=1.3.0

- scipy=1.11.3

- matplotlib=3.7.1

- seaborn=0.12.2

- tqdm=4.66.1

- tensorboard=2.14.0

- jupyter

- pip

- pip:

- torch-scatter

- torch-sparse
